# Variation of ewe olfactory secretome during a ram effect

**DOI:** 10.3389/fvets.2022.1033412

**Published:** 2023-01-09

**Authors:** Paul Cann, Chrystelle Le Danvic, Chantal Porte, Didier Chesneau, Matthieu Keller, Patricia Nagnan-Le Meillour

**Affiliations:** ^1^Université de Lille, CNRS, INRAE, Glycobiologie Structurale et Fonctionnelle, UMR8576, INRAE USC 1409, Lille, France; ^2^ELIANCE, Paris, France; ^3^INRAE/CNRS/Université de Tours/IFCE, Physiologie de la Reproduction & des Comportements, UMR 7247, INRAE 0085, Nouzilly, France

**Keywords:** male effect, olfactory secretome, Olfactory Binding Protein (OBP), salivary lipocalin, *O*-GlcNAc glycosylation, *Ovis aries*, proteomics

## Abstract

**Introduction:**

Under temperate latitudes, reproduction in *Ovis aries* displays a marked seasonality, governed by the photoperiod. In natural conditions, the transition between sexual rest and sexual activity in both sexes is induced by the decrease of day light. Meanwhile, specific odors emitted by a sexually active male are able to reactivate the gonadotropic axis of anovulatory ewes. This physiological effect is called “male effect”, precisely ram effect in the ovine species. We have previously shown that the secreted proteins, namely Olfactory Binding Proteins (OBP), contained in the nasal mucus constitute the olfactory secretome (OS), the composition of which is determined by the status of oestrus cycle of females and differs between sexual rest and sexual activity periods. The objective of this study was to test the hypothesis that exposure to sexually active male can also modify the composition of ewes olfactory secretome during a male effect, as well as hormones produced by the reactivation of the oestrus cycle in sexual activity period under natural conditions.

**Methods:**

We have set up a new non-invasive protocol of nasal mucus sampling and collected it from 12 ewes at different times during a ram effect. We analyzed the composition of their olfactory secretome by proteomics, mainly SDS-PAGE and MALDI-TOF mass spectrometry. As post-translational modifications of OBPs were a hallmark of ewes' sexual activity period, we were looking for glycosylation by western-blot and mass spectrometry.

**Results:**

The efficiency of male effect was low in stimulated ewes as only 3 females displayed elevated progesterone levels in their blood. Besides, half of control ewes (non-stimulated ones) were cycled. We noticed a common OS profile in ewes in anoestrus, versus OS of cycled ones. A very clear and important result was the apparition of *O*-GlcNAcylation, previously detected only in sexual activity, after only 30 min of male introduction into the flock.

**Discussion:**

This exploratory study paves the way for further experiments with larger flock to confirm and reinforce these results, and for eventually exploiting the nasal mucus as an indicator of females' receptivity to male odors.

## 1. Introduction

Due to the photoperiodic control of reproduction, female sheep do not ovulate during the anoestrus or sexual rest period under temperate latitudes. By contrast, when the photoperiod is favorable, ovarian cycle can occur (1). Under natural conditions, a reactivation of the gonadotropic axis during the season of sexual rest can be induced by the presence of a sexually active ram in a flock of ewes, this effect being known as the male effect, or ram effect in the ovine species, as described in 1944 by Underwood et al. (2). The higher efficiency of the male effect is observed during the early or late stages of the sexual rest season when the anoestrus state is not too deep. Ewes can respond to the male effect in two different ways, characterized by the same modalities but with different delays in the cycle reactivation (3–5). In both cases the introduction of a sexually active male in a flock of anoestrus ewes reactivates the luteinizing hormone (LH) pulsatility (6, 7) that increases over time until a preovulation peak leading to a silent ovulation (not associated with heat behavior) within 3 days. In a first case, the *corpora lutea* formed by ovulation is secreting progesterone (P4) during around 17 days (normal duration of cycle) until a second ovulation occurs, associated this time with heat behavior. In the second case, the luteolysis occurs after 4–5 days of contact (8) and a second silent ovulation appears 6 days after the first ovulation (short cycle), followed by a third ovulation 17 days later (23 days after the first ovulation), which is associated with heat behavior. These two cases lead to two waves of lambing with a 6-days gap (2). The occurrence of each case depends on the depth of the anoestrus that is indirectly estimated by the number of females with spontaneous ovulation before male introduction. The effectiveness of the male effect depends on many factors such as experience of the female (9), its age, its metabolic state or the level of ram sexual behavior (10). Vocal and visual cues from the male can only induce a low LH pulsatility, unable to lead to a complete reactivation of the gonadotropic axis in females (11, 12). By contrast, the ram odor is the key factor. Indeed, exposure to the wool, which is the main source of male odor, strongly stimulates the LH pulsatility (13, 14) leading to its reactivation and ovulation. Such a mixture of molecules capable to deeply modify the female physiology can be classified as a primer pheromone, according to the definition of Karlson and Lüscher (15). Although some compounds of the putative pheromonal mixture involved in the ram effect have been identified (16), the identification of all the chemical components remains challenging.

When moving at the neurobiological level, the circuit involved in the processing of male odor has been explored. For example, olfactory bulbectomy (lesion of both the main and accessory olfactory systems) as well as lesion of the main olfactory system induces a major loss in the response of the females to the male odor, in contrary to the lesion of the vomeronasal organ (11, 14, 16). Regarding the more central steps, results derived from the use of c-Fos as a marker of cellular activation have given a map of the neural pathways involved in the effect of the ram fleece on the LH secretion in anoestrous ewes, confirming the critical role of the main olfactory system. Indeed, the main olfactory bulb and the cortical nucleus of the amygdala are specifically more activated by the male than the female odor and seem therefore to be primarily involved in the detection and the integration of the ram odor from the fleece (14). Thus, male pheromones can be detected by the main olfactory system and/or the accessory ones as it has been shown for several species [reviewed in Lévy and Keller (17)]. Besides central processing, very few is known about the role of the peripheral step of olfactory system in the male effect.

In a previous work, we have explored the possibility that perireceptor events of the olfactory system, in particular Olfactory Binding Proteins (OBP) that compose the olfactory secretome (OS) of the nasal mucus, could also be subjected to plasticity between sexual rest and sexual activity period (18). Indeed, variation of the pig OS according to sex and hormonal status (before and after puberty) have suggested a possible hormonal control of OBP secretion throughout life (19). Moreover, it has been proposed that the OS could be a phenotype reflecting the capability of odors detection and could be under control of endogenous factors such as hormones (19). The OS is composed of around 30 isoforms in pig (19, 20), 50 in goat and between 20 and 30 in ewes (18) generated by post-translational modifications (PTM) from a reduced number of primary OBP sequences. We have shown that the hormonal changes linked to the alternation of sexual rest and sexual activity periods have a high impact on the composition of OS of ewe and goats (18). In ewe, there is an increasing complexity of the secretome in sexual activity (SA) period, in term of primary sequences expressed and/or in their PTM, generating more isoforms. We hypothesized that this increased number of isoforms could correspond to an adaptation of the peripheral level of olfactory system to enhance the capability of detection for male odor, since PTM, such as phosphorylation (21) and *O*-GlcNAcylation, a particular glycosylation, drive the binding properties of OBP in pig (22, 23). In particular, the sexual activity period of ewe is characterized by the expression of salivary lipocalin (SAL) and *O*-GlcNAcylation of OBP, totally absent in sexual rest (SR) period. This marked difference questions about the influence of ram odor on the composition of ewe OS during a male effect, which induces the reactivation of the gonadotropic axis and its release of hormones. We formulated two hypotheses for the impact of male odor detection, which could lead to either a transitional OS, then to the sexual activity OS, or to a specific OS, typical of the response to male stimulation. We report herein the variation of ewe olfactory secretome during a male effect, with special attention to new proteins expression and their post-translational modifications.

## 2. Results and discussion

### 2.1. Physiological status of females by progesterone level monitoring

In breeding systems, the male effect is performed in order to extend the reproduction period and a reactivation of the ovarian cycle supposes that this cycle is blocked in seasonal anoestrus. Except for ewes CTRL2 and CTRL3 that belong to the control group, the anoestrus status at the beginning of the male effect protocol was assessed for all other ewes (Table 1). Indeed, their P4 concentration was under the detection threshold (0.25 ng/mL) at least 1 month before exposure to the male.

**Table 1:** Monitoring of progesterone concentration in ewe's blood every week before exposure to the male.

		**Days before male effect protocol (T0)**					
		−**34 d**	−**27 d**	−**20 d**	−**16 d**	−**13 d**	−**6 d**
	**Progesterone (ng/mL)**						
Batch 1 stimulated	STIM1 (30056)	< 0.25	< 0.25	< 0.25	< 0.25	< 0.25	< 0.25
	STIM2 (30094)	< 0.25	< 0.25	< 0.25	< 0.25	< 0.25	< 0.25
	STIM3 (30118)	< 0.25	< 0.25	< 0.25	< 0.25	< 0.25	< 0.25
Batch 1 control	CTRL1 (30140)	< 0.25	< 0.25	< 0.25	< 0.25	< 0.25	0.9
	CTRL2 (30225)	3.1	< 0.25	1.1	3.4	3.6	0.6
	CTRL3 (30228)	1.0	1.0	1.1	1.3	1.1	1.3
Batch 2 stimulated	STIM4 (40068)	< 0.25	< 0.25	< 0.25	n. a.	< 0.25	< 0.25
	STIM5 (40095)	< 0.25	< 0.25	< 0.25	n. a.	< 0.25	< 0.25
	STIM6 (40161)	< 0.25	0.5	0.5	n. a.	< 0.25	< 0.25
Batch 2 control	CTRL4 (40088)	0.5	< 0.25	< 0.25	n. a.	< 0.25	< 0.25
	CTRL5 (40163)	< 0.25	< 0.25	< 0.25	n. a.	< 0.25	< 0.25
	CTRL6 (40201)	< 0.25	< 0.25	< 0.25	n. a.	< 0.25	< 0.25

Animals are referenced with their national number in brackets.

The monitoring of P4 during the male effect protocol brought very interesting information about females' hormonal status. Results (shown in Supplementary Table 1) revealed that half of the stimulated ewes responded to the male (STIM1, STIM2, STIM3 that were grouped in the same batch), whilst ewes STIM4, STIM5, and STIM6 of the second batch displayed minor increase of P4 level, indicating that exposure to the male was unable to reactivate their cycle. Interestingly, these 3 ewes were 1 year older than the “responding” ewes of the first batch. Besides, the older ewes of the second control batch were in anoestrus, together with the younger CTRL1 ewe of the first control batch (Supplementary Table 2). The ewes that were not in anoestrus before the protocol (CTRL2 and CTRL3) were detected as cycled. Indeed, it has been reported that, depending on the breed, some ewes can display heat, and even silent ovulation during anoestrus in April/May, together with upper P4 levels than expected (24). These results have been faced to the biochemical data of OS composition below. As none of the older ewes responded to the exposure to the males, we have focused our analyses of OS by proteomics on younger ewes (STIM1, STIM2, STIM3, CTRL1, CTRL2, and CTRL3).

### 2.2. Variation of olfactory secretome profile according to male-female contact

The separation of total soluble proteins of the nasal mucus was first performed by 2-dimensional electrophoresis (Supplementary Figure 1) to compare the profiles with those of sexual rest (SR) and sexual activity (SA) periods previously described in Cann et al. (18). Indeed, ewes STIM1, STIM2, and STIM3 of the stimulated batch were already analyzed in SA and SR. For these 3 females and the 3 control ewes, the distribution of proteins followed the same pattern in two strings of protein spots at 20 and 17 kDa apparent mass, but the third string typical of SA (18) did not appear in any of the ewes exposed or not to the male. As no differences were highlighted by 2D-E between stimulated females and controls, or between sample times for a same female, we performed the analysis of ewes OS by one-dimension SDS-PAGE (1D).

#### 2.2.1. Is there an expression of new protein(s) in response to the perception of male odor?

**Figure 1:**
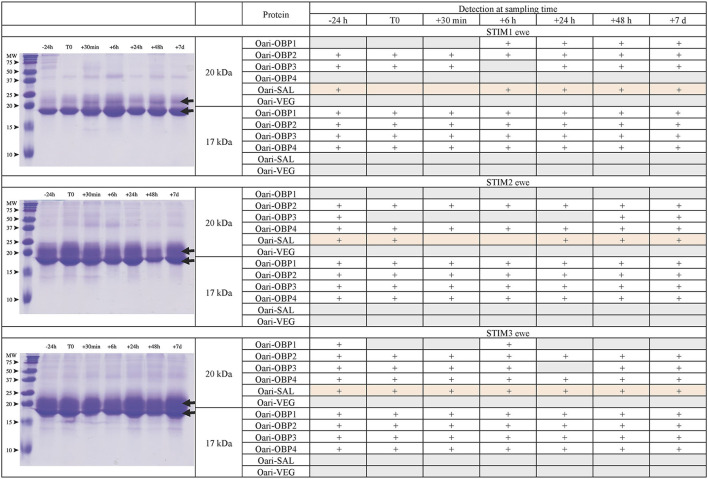
Analysis of olfactory secretome of ewes exposed to the male. **(Left)** One-dimension separation (SDS-PAGE) of total soluble proteins extracted from nasal mucus at different sampling times. Coomassie blue staining. Molecular weight marker, Precision Plus Protein Standard Dual Color (Bio-Rad). Arrows indicate the 17 and 20 kDa migrating bands, the protein composition of which was determined by MALDI-TOF MS and is given in tables on the **(Right)**.

**Figure 2:**
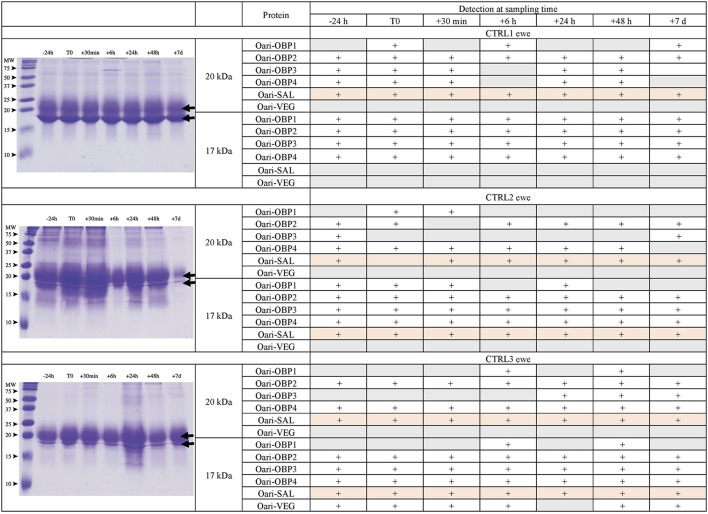
Analysis of olfactory secretome of control ewes. **(Left)** One-dimension separation (SDS-PAGE) of total soluble proteins extracted from nasal mucus at different sampling times. Coomassie blue staining. Molecular weight marker, Precision Plus Protein Standard Dual Color (Bio-Rad). Arrows indicate the 17 and 20 kDa migrating bands, the protein composition of which was determined by MALDI-TOF MS and is given in tables on the **(Right)**.

**Figure 3:**
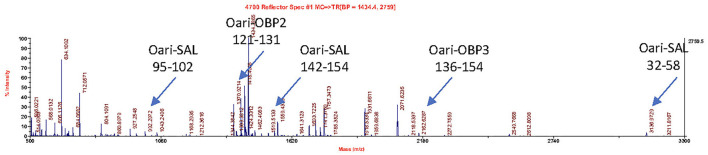
MALDI-TOF spectrum of peptides resulting from the trypsin action on the 20 kDa band of the olfactory secretome of CTRL2 ewe (sampling time + 7 d). Arrows indicate *m/z* corresponding to Oari-OBP2, Oari-OBP3, and Oari-SAL peptides (listed in Supplementary Table 12). The position in the sequence is indicated below.

Figures 1, 2 summarize the results obtained for the younger ewes, exposed or not to the male, respectively. Identification of proteins was performed on spots excised from each band of SDS-PAGE gels by MALDI-TOF mass spectrometry after trypsin digestion (Bottom-up mass spectrometry). MALDI-TOF MS results were manually processed from the chromatograms of obtained spectra as this manner is more accurate than identifying peptides from the peak list, which does not give any information on the quantity and thus could lead to misinterpretation of presence/absence of a protein. Figure 3 gives an example of such a chromatogram obtained from CTRL2 ewe at sampling time day 7. Measured masses of resulting peptides were compared to those of theoretical maps (https://web.expasy.org/peptide_mass/) coming from trypsin digestion of the main components of ewe OS, which are Oari-OBPs, -SAL, and -VEG (Supplementary Tables 3–14). In all ewes, the OS is composed of Oari-OBP1 (Uniprot# W5PHM2), Oari-OBP2 (QEY02201.1), Oari-OBP3 (W5PGW3) and Oari-OBP4 (QEY02202.1) sequences, retrieved in the 17 and 20 kDa bands. The sequences coverage was between 4.48 and 36.70%.

Interestingly, CTRL2 and CTRL3 ewes, which appeared cycled, displayed the same profile with a more intense band at 20 kDa than at 17 kDa. Conversely, stimulated ewes, and CTRL1 ewe in anoestrus, displayed a more intense 17 kDa band than the 20 kDa ones. This later profile seems to be typical of anoestrus ewes, whatever they have been exposed or not to the male. Indeed, the composition of the two protein bands is also similar in these 4 ewes. In particular, there are more Oari-OBP2 peptides in the 17 kDa band of these ewes, and more Oari-OBP4 in this band in cycled ewes CTRL2 and CTRL3. The OS of stimulated ewes and CTRL1 ewe is also characterized by the lack of Oari-SAL peptides in the 17 kDa band, although such peptides were retrieved in the 17 kDa band in the cycled CTRL2 and CTRL3 ewes. The presence of SAL in anoestrus ewes is not totally in accordance with our previous work (18). Indeed, the ewes exposed to males in this work were previously included into the comparison of OS between SR and SA in a previous work (18). In STIM2 and STIM3 ewes, Oari-SAL was not identified in 2D spots of sexual rest olfactory secretome, so we have suggested that Oari-SAL expression could be triggered by hormones in sexual activity period. In order to better understand this discrepancy, we amplified SAL-like sequences from nasal tissue of CTRL1 ewe in season to ascertain the proteomics data and the presence of SAL sequences in the ewe secretome. Two different sequences of Oari-SAL were expected from the Ensembl database of sheep genome, Oari-SAL1 (W5P8W4) and Oari-SAL2 (W5P8Y1), and their peptides were identified by mass spectrometry in sexual activity period (18). Ten clones containing amplicons resulting from touchdown PCR revealed 6 different SAL-like sequences (Blastp) with mutations at 18 positions in the translated protein sequence (Supplementary Figure 2). Theoretical maps of trypsin digests of the six SAL sequences were obtained (https://web.expasy.org/peptide_mass/) to search for discriminant peptides in MALDI-TOF analyses of ewes OS. This diversity, although interesting, complexified the interpretation of presence/absence of SAL during the male effect. Facing this multiplicity of sequences, a question raised about the accuracy of Oari-SAL absence in previous SR analyses (18). Indeed, at the time of this previous work, we had not yet amplified the 6 different Oari-SAL sequences and did not include them in the query to the software search used with Nano-LC-MS/MS data (Mascot, Matrix Science, London). In the present work, Oari-SALs were identified with 10 peptides of different *m/z*, and for each analysis, at least one was part of the Oari-SAL1 and/or OariSAL2 of Ensembl database, meaning that even without the new sequences, SAL would be identified. New sequences were deposited in Genbank database under accession numbers MT921827 and MT921828.

These data evidenced differences between cycled ewes (CTRL2 and CTRL3) and ewes in anoestrus at the beginning of the ram effect protocol (stimulated ewes and CTRL1 ewe), and confirmed the impact of hormonal status on the ewes olfactory secretome. But no particular event triggered by the male odor perception appeared at the level of olfactory protein expression.

#### 2.2.2. Is there a change in post-translational modifications during the male effect?

The detection of peptides of the same protein in bands at 17 and 20 kDa denotes the presence of isoforms of these proteins, which could come from multiple phosphorylation events (mass increments of 79 Da), and/or GlcNAcylation (mass increments of 203 Da), and/or *N*-glycosylation (multiple of 204 Da), explaining (1) differences in apparent molecular masses for the same protein sequence, and (2) the absence of detection of potentially modified peptides, reflected in the low percentages of sequence coverage (between 4 and 37%, Supplementary Tables 3–14). Indeed, many potential sites of phosphorylation, *O*-GlcNAcylation, and *N*-glycosylation were predicted by *in silico* analysis for OBPs of the ovine olfactory secretome (Table 2, detailed in Supplementary Figure 3), and could be differently occupied during animal's life. Although numerous phosphorylation sites were predicted for all Oari-OBPs, -VEG, and -SAL (Table 2), we do not report here the search for phosphorylation, as no difference between SA and SR periods have been detected previously (18).

**Table 2:** Number of sites of post-translational modifications predicted for OBPs of ewe olfactory secretome.

**Protein**	**Accession number**	**Number of amino acids**	**Number of predicted sites**		
			***N*** **-glycosylation**	**Phosphorylation**	***O*** **-GlcNAcylation**
Oari-OBP1	W5PHM2	155	0	14	1
Oari-OBP2	QEY02201.1	158	1	10	4
Oari-OBP3	W5PGW3	154	1	17	2
Oari-OBP4	QEY02202.1	156	0	11	2
Oari-SAL1	W5P8W4	167	4	10	2
OariSAL2	W5P8Y1	167	5	14	4
Oari-VEG	W5NUS5	157	0	9	6

##### 2.2.2.1. Analysis of the *N*-glycosylation of Oari-OBP(s) and -SAL(s)

In contrast, *N*-glycosylation was described as one of the hallmarks of sexually active ewes OS (18). Thus, the *N*-glycosylation profile of ewes OS included in this study were analyzed by reflectron positive ion mode MALDI-TOF mass spectrometry. After their release by enzymatic digestion, *N*-glycans were derivatized, allowing methylation of the hydroxyl and acetyl groups. The search for *N*-glycans was run on samples coming from 5 different ewes and this choice was determined by the quantity of samples remaining for each ewe. For the “true” control ewe CTRL1, and ewes STIM4 and STIM6, which have not responded to the male stimulation, no signal at all could be detected in their OS sample as expected (data not shown). For the analysis of stimulated ewes STIM1 and STIM3, the OS samples were pooled at each sample time to increase the sensibility (e.g., STIM1 + STIM3 at −24 h, STIM1 + STIM3 at T0, and so on). Some peaks were detected but their *m/z* values did not match with any *N*-glycan structure (Supplementary Figure 5). We could conclude that no *N*-glycosylation of OS proteins was induced by exposure to male odor.

**Figure 4:**
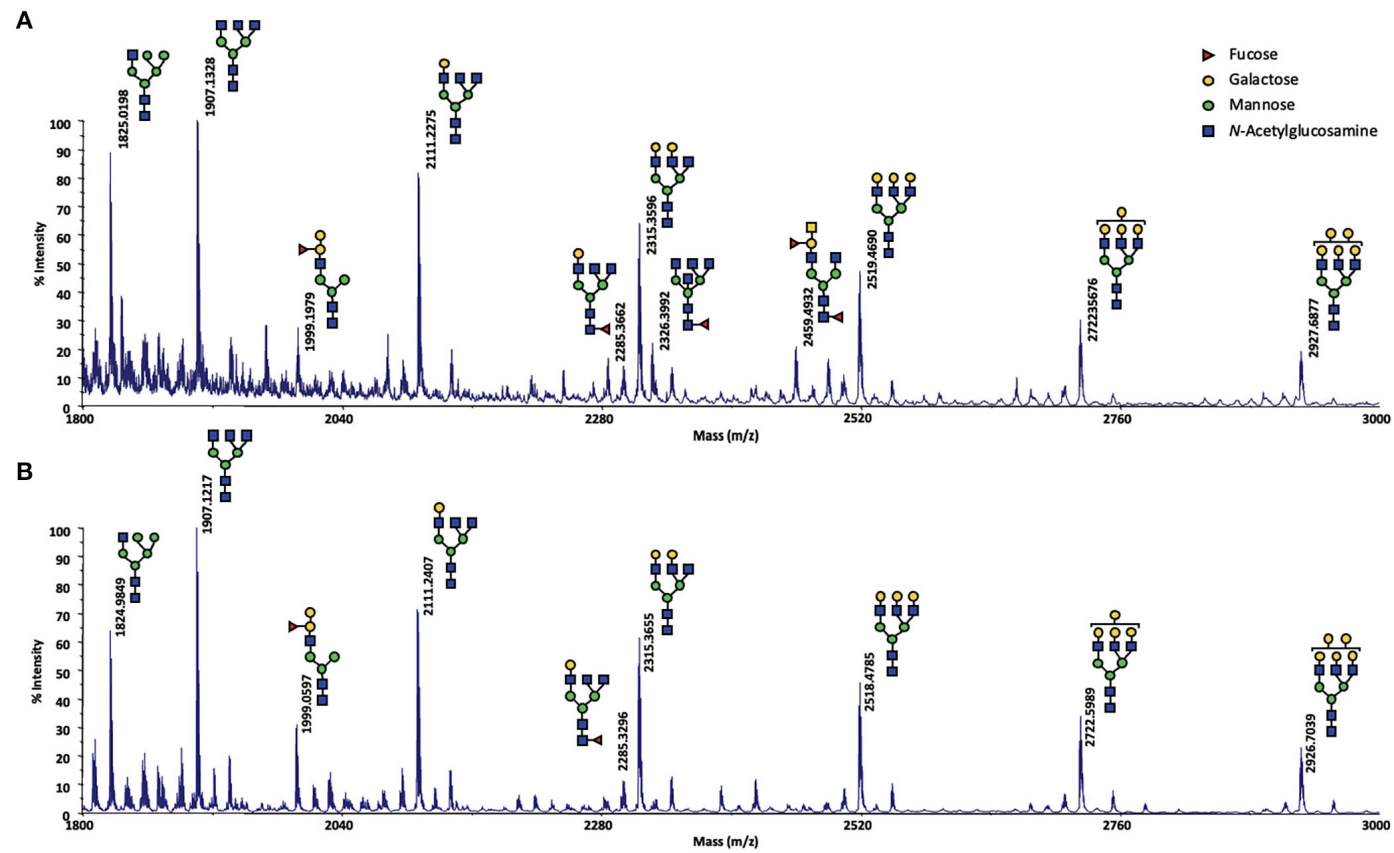
MALDI-TOF spectrum of permethylated N-glycans extracted from olfactory secretome of the CTRL2 ewe during the sexual activity period **(A)** in 2017; **(B)** in 2018. N-glycans were detected as sodium adduct ions [M + Na]^+^ over a mass range of *m/z* 1,800–3,000. The presence of structural isomers cannot be excluded. Structural schemes of glycans are depicted following the CFG notation: *N*-acetylglucosamine (blue square), fucose (red triangle), mannose (green circle), galactose (yellow circle).

Meanwhile, as the results of this analysis were crucial to understand the effect of the exposure to the male on ewes OS, they had to be confirmed. For that, MALDI-TOF MS profiles of OS of CTRL2 ewe, collected during the sexual activity period in 2017 and 2018 (year of the male effect protocol described here) were analyzed in exactly the same conditions. The structures shown in Figure 4 were assigned from compositional information inferred by their *m/z* values. The presence of structural isomers cannot be excluded as no MS/MS analysis could be performed, due to low sample quantity. The obtained spectra proved to be informative for the repeatable detection of 9 and 11 glycans during the sexual activity (SA) period for year 2018 and 2017, respectively (Figure 4). Except one hybrid structure (*m/z* 1825, HexNAc_3_Hex_5_), all the observed *N*-glycans were found to be complex structures. A distinctive series of signals cluster, which is related by a unique increment of 204 amu, corresponding to a Hex (hexose) difference, was characterized at *m/z* 1907 (HexNAc_5_Hex_3_), 2111 (HexNAc_5_Hex_4_), 2315 (HexNAc_5_Hex_5_), 2519 (HexNAc_5_Hex_6_), 2723 (HexNAc_5_Hex_7_) and 2927 (HexNAc_5_Hex_8_). Two other complex N-glycans at *m/z* 1999 (FucHexNAc_5_Hex_5_) and 2285 (FucHexNAc_5_Hex_4_) completed the profile. For the year 2017, two additional fucosylated glycans could be observed at *m/z* 2326 (FucHexNAc_6_Hex3) and *m/z* 2459 (Fuc_2_HexNAc_5_Hex_4_). The difference between the 2 years could easily be explained by a sampling at different times of the cycle during the SA period, as we have not precisely followed the oestrus cycle during this period. So, sampling could have been performed either in pro-oestrus, or oestrus, or di-oestrus. Over the 2 years, very similar *N*-glycan profiles were observed, confirming that *N*-glycans seem to be specific to the sexual activity period. Their presence during SA period would contribute to the complexification of the OS highlighted during this period in our previous work (18).

Because of a limited quantity of proteins, the whole OS was profiled for its content in *N*-glycans, but it was not possible to study each protein separately. Therefore, the detected structures could not be assigned to one or another protein sequence of the OS. Indeed, *N*-glycosylation of Asn residues was predicted for Oari-OBP2 (1 site), Oari-OBP3 (1 site), Oari-SAL1 (4 sites) and Oari-SAL2 (5 sites) (Table 2). Similar structures have been described for porcine SAL (25), but never for OBPs *stricto sensu*. This kind of glycosylation is the oldest ones reported and is typical of the secretion pathway (26). It is thus intriguing that porcine OBPs, although secreted and coming from the secretion pathway are not *N*-glycosylated, but modified by an atypical glycosylation, *O*-GlcNAcylation, as well as ovine OBPs (18).

##### 2.2.2.2. *O*-GlcNAcylation of Oari-OBP(s)

**Figure 5:**
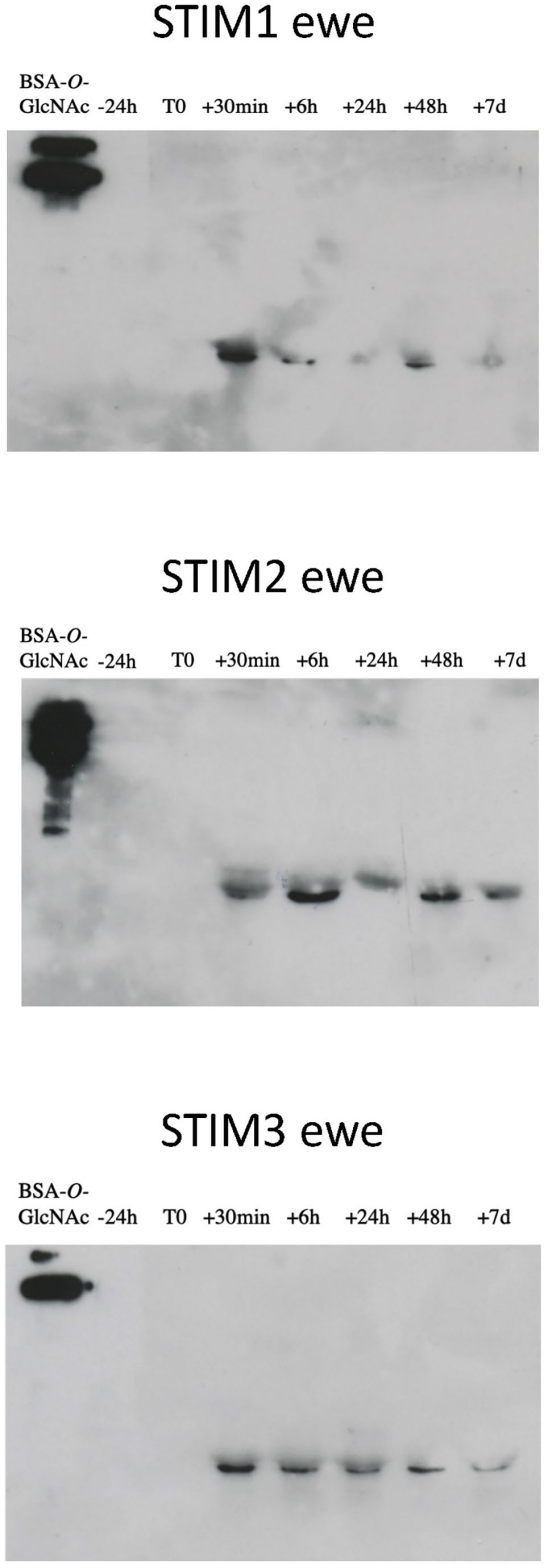
Immunodetection of *O*-GlcNAcylated proteins in ewes exposed to the male. Western-blot with CTD110.6 antibody (1/5,000) on proteins of nasal mucus (15 micrograms per well) sampled during a ram effect. ECL detection, 10 min exposure.

**Figure 6:**
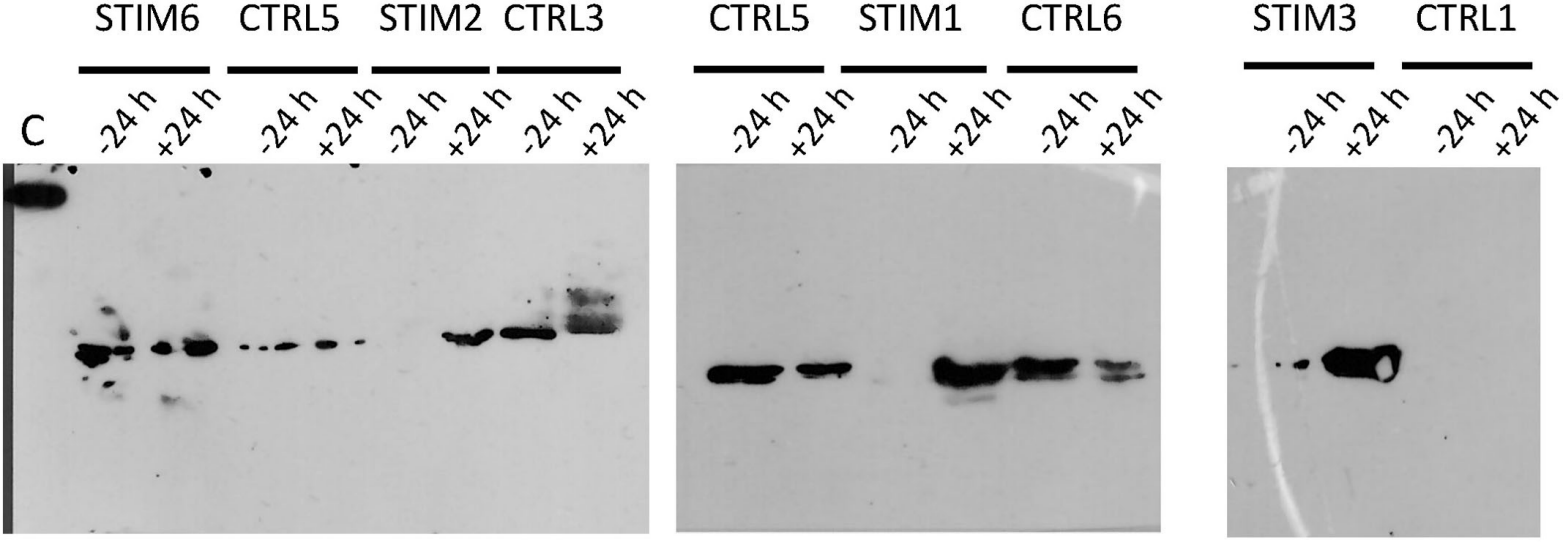
Immunodetection of *O*-GlcNAcylated proteins by western-blot with CTD110.6 antibody in olfactory secretome of ewes, stimulated or not by male. Sample times (−24 h and +24 h) frame the time of male introduction (T0). C, control; 5 ng of BSA-GlcNAc.

Indeed, one of the markers of sexual activity previously identified was the presence of *O*-GlcNAcylation in the secretome of ewes (18), which was totally absent in sexual rest. This was confirmed by further experiments in additional animals (data not shown). We have thus performed immunodetection of *O*-GlcNAc by western-blot with CTD110.6 antibody, specific to secreted proteins *O*-GlcNAcylation (27). This specificity was checked by competing the link antibodies/antigens (here GlcNAc linked to OBPs) by incubation between free GlcNAc and CTD110.6 antibody, prior to incubation with the membrane after electroblotting (Supplementary Figure 4A). In addition, to overcome the possibility that CTD110.6 could label *N*-glycan chains (although not detected in this study), samples of ewe STIM1 were first treated with PNGaseF to remove eventual *N*-Glycan chains, and then incubated with CTD110.6 (Supplementary Figure 4B). Both controls demonstrated that CTD110.6 specifically labeled *O*-GlcNAcylated proteins, and not *N*-glycans. Interestingly, *O*-GlcNAc signal appeared as soon as 30 min of contact with the male for the three stimulated ewes (Figure 5). This short answer suggests that the action of male odors on ewes' brain is very quick, triggering the reactivation of the OBP *O*-GlcNAcylation, and thus, expression of new isoforms. As the number of aliquots of samples was restricted, we performed western-blot with CTD110.6 on two samples collected at −24 h and +24 h, that frame the apparition of GlcNAc for three stimulated ewes, which were included for comparison (Figure 6). The results were confirmed for the three responding ewes, and no signal was detected for the true control CTRL1 ewe. The cycled CTRL3 ewe showed a GlcNAc signal in accordance to its hormonal status. Conversely, the ewes STIM6, CTRL5, and CTRL6 displayed *O*-GlcNAc signal at any time. It was surprising and contradictory to their low P4 values indicating an anoestrus. We were expected an absence of *O*-GlcNAcylation.

The appearance of GlcNAc signal after 30 min exposure to the male is in agreement with the physiological events that occur after male stimulation. The short-term response, within few minutes, is characterized by the secretion of GnRH, which induces the increase of LH pulses in intensity and frequency (7, 28) until the pre-ovulatory peak after 24–48 h of contact. In response, ovaries trigger ovulation (long-term response). At the olfactory system level, it seems that reactivation of the gonadotropic axis leads to the reactivation of *O*-GlcNAcylation pathway in olfactory tissue secreting OBPs, as early as 30 min after male exposure.

## 3. What does specific isoforms expression mean in response to male exposure?

To return to our starting hypotheses, and to discuss our data with the caution linked to a small number of animals relevant to the study, we did not observe any gradual transition between SR and SA phenotypes, but more likely a “male effect” phenotype characterized by the reactivation of OBP *O*-GlcNAcylation, under endocrine control, probably LH stimulation. Our previous studies have demonstrated the fine regulation of OBPs binding properties to pheromonal components by post-translational modifications, especially phosphorylation and *O*-GlcNAcylation in pig (23). After exposure to the male and its odor, the ewe olfactory system seems to express specific *O*-GlcNAcylated isoforms, possibly adapted to the binding with molecules specifically secreted by the sexually active male, which remain to be identified.

This is the first study dealing with the impact of sexually active male exposure on ewes' olfactory equipment, especially at this molecular level. The monitoring of nasal mucus composition was rendered possible by the setup of a non-invasive sampling protocol that could easily be adapted to other crucial events in animals' life in relation to odor perception. The low efficiency of male to stimulate females reflects the reality of this practice but reduced the size of the flock and weakened our results. Nevertheless, the analysis of olfactory secretome was strengthened by high-resolution methods. Further experiments are needed to confirm the data, particularly by extending the number of animals. Finally this is not sure that a high number of females would change the main result, that is OBP *O*-GlcNAcylation. Besides, these results have established a link between hormonal status and OS composition, indicating that OS could eventually be used as a marker of females' receptivity to the ram odor.

## 4. Methods

### 4.1. Animals and ethical statements

Animals (*Ovis aries*, Ile de France, 3–5 years old) were bred at “Unité Expérimentale de l'Orfrasière” (UEPAO, INRAE, France; doi.org/10.15454/1.5573896321728955E12). The male effect protocol described in this work was performed in April 2018, at the beginning of seasonal anoestrus. Measurement of progesterone (P4) concentrations were run on blood samples before and during male exposure to ascertain females' anoestrus and response to the male. Meanwhile, to avoid any bias in results interpretation, we worked in a blind manner, which means that P4 levels in ewes' blood samples were not known when biochemical analyses of the OS were performed. Over the entire experimental period, ewes were maintained indoors in large pens (6 m × 4 m for 6 animals) under natural photoperiod conditions. Both groups (stimulated and control) were housed in different barns spaced by more than 100 m. Animals were fed daily with dehydrated lucerne, maize, straw, and a supplement of vitamins and minerals, and they had free access to water, according to INRAE standards for maintaining adult weight and for providing adequate nutrition. The ram effect protocol, non-invasive sampling of nasal mucus and blood sampling for determination of progesterone concentration were validated by ethical committee n° 019 of French “Ministère de l'Enseignement Supérieur, de la Recherche et de l'Innovation” (APAFIS#13067–2018011616242285 v2).

### 4.2. Male effect protocol and nasal mucus sampling

Rams were submitted to a photoperiodic treatment of long days (16 h light and 8 h dark) from November to January 15th. This treatment was provided in a light proof building and the intensity of the artificial light provided was at least 300 lux at the level of animal eyes. From January 16th to the end of the study, the light treatment was stopped and the bucks were moved to inside barns exposed to natural light, therefore natural photoperiodic conditions. They received additional subcutaneous melatonin implants (Melovine, CEVA, France; 18 mg). This treatment stimulates the male reproductive activity during the non-breeding season after 45–60 days.

Twelve anovulatory ewes were maintained in two separate buildings, each group of 6 ewes were separated into two batches. For each of the two “stimulated” batches, one sexually active male was introduced at day 0 and replaced by another male every day until day 7 (5). Previously, rams were sexually activated by photoperiodic treatments and melatonin implants (1). During the protocol, no reproduction could occur thanks to a leather apron on rams that prevented penetration. The control ewes remained isolated from rams throughout the male effect protocol in a separate building far from the stimulated ewes and rams.

Non-invasive nasal mucus sampling was made by gently wiping the nasal mucosa with a sterile gauze (5 cm × 5 cm, Laboratoires Mercurochrome) twisted 5 times in each nostril. Each gauze was put in 20 mL glass vial and stored à −80°C until protein extraction. The nasal mucus sampling was made 24 h and few minutes (T0) before exposure to the male, then the sampling was made after the male-female contact at 30 min, 6 h, 24 h, 48 h and at day 7 after male introduction into the flock. The physiological status of females was checked by blood progesterone monitoring every week starting 1 month before the male exposure (Table 1) then every day from J−1 to J+9 (Supplementary Tables 1, 2). In the non-stimulated batches (control groups) nasal mucus and blood were collected at these same time points. All blood samples were collected by jugular venipuncture in 5-mL tubes containing 30 mL heparin. Plasma was obtained after centrifugation 30 min at 3,000 g and 4°C, then stored at −20°C until hormonal dosage. Progesterone concentration was determined by using a direct RIA method (29). Sensitivity of the assay was 0.25 ng/mL. Females with progesterone concentrations >1.0 ng/mL were considered to have ovulated and therefore not to be in anoestrus (4).

### 4.3. Molecular cloning of SAL-like sequences

The CTRL1 ewe was sacrificed in sexual activity period with practices in agreement with EU directive 2010/63/EU. The nasal mucosa was resected immediately after death in the ISO9001-certified slaughterhouse of INRAE experimental farm UEPAO (Nouzilly, France) according to the agreement E37-175-2. Total RNA was extracted from 30 mg of tissue with the RNeasy Mini kit (Qiagen). RNA (5 mg) was used to amplify 5′and 3′ ends by RACE PCR (18) followed by reverse transcription with the SuperScript™ IV kit (Invitrogen) according to the supplier protocol. The RT template was used in touchdown PCR (Invitrogen protocol, 18) to amplify *OariSAL* with the primers: *5OariSAL* 5′-ACCCACATGAAGCTGCTGCTGCTGTGTCT-3′, and *3OariSAL* 5′-AAGACAACTAGG CCACTCCATTCCCTCGC-3′. The PCR product was cloned into pCR4^®^-TOPO plasmid (TOPO^™−^TA cloning™ kit, Invitrogen). After amplification in *Escherichia coli* One-Shot Top 10 chemically competent cells (Invitrogen), the recombinant plasmids were purified (QIAprep Spin Miniprep kit, Qiagen) and sequenced in both senses (Eurofins genomics).

### 4.4. Protein extraction

Chemicals were purchased at Sigma-Aldrich, unless specified. Proteins were extracted from the gauzes as described in Cann et al. (18) by phase partition with cold chloroform/methanol solution (2/1, v/v). Two successive extractions were performed from the gauzes, first with an incubation with 4 mL of chloroform/methanol at 4°C for 1 h 30, then 1 mL of MilliQ water was added and gauzes were squeezed with a sterile syringe. The second extraction was performed by adding to the same gauze 2 mL of chloroform/methanol at 4°C and 0.5 mL of MilliQ water, then the gauze was squeezed with another sterile syringe. The liquids recovered from the two extractions were pooled and centrifuged at 3,234 rcf for 20 min at 4°C. The methanol phase was collected (c. a. 4 mL) then washed once with the same volume of chloroform before another centrifugation as described above. Methanol phases were collected and dried in a vacuum concentrator [Eppendorf Vacufuge Plus Concentrator (RRID:SCR_019876)]. Proteins were aliquoted by 30 and 15 μg after quantity estimation by SDS-PAGE.

### 4.5. Two-dimensional electrophoresis

For 2-DE, dried proteins were resuspended in 10 μL water, then in 120 μL of rehydration buffer as already described. Immobiline Dry Strips (IPG strips: pH 3–5.6, 7 cm, GE Healthcare or pH 4–7, 7 cm, Bio-Rad) were submitted to passive rehydration with samples during 16 h at room temperature (RT). First dimension (isoelectrofocalisation) was performed on PROTEAN i12™ IEF system (BioRad), with the standard program “7-cm Gradual S-1” (250 V rapid for 30 min, 1,000 V gradual for 1 h, 5,000 V gradual for 2 h and a hold of 5,000 V), with a current limited to 50 μA/gel. When IEF was complete (9,000 VH final), IPG strips were incubated successively for 15 min in the equilibration buffer (375 mM Tris-HCl pH 8.8, 6 M Urea, 2% (w/v) SDS and 30% (v/v) glycerol) containing first 1.5% (w/v) DTT then 2% (w/v) iodoacetamide. The second dimension was performed using 16.8% acrylamide SDS-PAGE in Mini-PROTEAN^®^ Tetra cell (Bio-Rad) with Precision Plus Protein Standard Dual Color (Bio-Rad) as molecular mass markers.

### 4.6. Analysis of proteins by mass spectrometry

Proteins (30 μg) were separated by SDS-PAGE, then stained with colloidal Coomassie blue R solution (19). Spots were excised from the main bands, then unstained overnight at 4°C in 50% ACN/50 mM ammonium bicarbonate (v/v). Disulphide bridges were reduced with 10 mM DTT at 56°C for 1 h, then alkylated with 50 mM iodoacetamide in 50 mM ammonium bicarbonate at room temperature (RT) for 45 min in the dark. Before trypsin digestion, spots were washed with 50 mM ammonium bicarbonate and dehydrated with 100% ACN. Spots were incubated overnight with 100 ng of Trypsin Gold (Promega) in 50 mM ammonium bicarbonate at 37°C. Peptides were extracted from the gel by two incubations in 10% formic acid/45% ACN at 30°C for 15 min, then with 5% formic acid/95% ACN at RT for 10 min. Peptides were desalted by centrifugation in Pierce^®^ C18 spin column (Fisher Scientific) according to manufacturer's instructions. Peptides were solubilised in 0.1% trifluoroacetic acid and mixed with 10 mg/mL α-Cyano-4-hydroxycinnamic acid matrix (Sigma-Aldrich). Matrix Assisted Laser Desorption Ionization—Time Of Flight Mass spectrometry (MALDI-TOF MS) analysis was performed on a Voyager DE Pro mass spectrometer [ABI Voyager DE Pro MALDI-Mass Spectrometer (RRID:SCR_019340)]. The instrument was used in positive reflector mode, measuring peptide masses on a range of 500–4,000 Da. Spectra were analyzed with Data Explorer V4.6 (Applied Biosystems). Raw data (.t2d files) were processed by using DataExplorer^®^ software (ABSCIEX).

### 4.7. Immunodetection of PTM by western-blot

Aliquots of 15 μg of total protein were separated by SDS-PAGE (19), then electroblotted with Trans-Blot Turbo Transfer System (Bio-Rad) onto PVDF membrane (Trans-Blot Turbo Transfer Pack PVDF Mini, Bio-Rad). For detection of *O-*GlcNAcylated proteins, the membrane was incubated 1 h at RT in blocking buffer (3% BSA in PBS-T, Phosphate Buffered Saline, 0.1 M sodium phosphate, 0.15 M NaCl, 0.05% Tween 20 v/v, pH 7.2), then with the primary antibody CTD110.6 (Sigma-Aldrich Cat# O7764, RRID:AB_1079524) at 1:5,000 in 3% BSA/PBS-T at RT for 1 h. The membrane was washed 6 times in PBS-T before secondary antibody incubation 1 h at RT (Sigma-Aldrich Cat# A8786, RRID:AB_258413) at 1:30,000 dilution. The signal was revealed by chemiluminescence with the SuperSignal™ West Dura Extended Duration Substrate (Pierce, Fisher Scientific). The specificity of CTD110.6 antibody was assessed by a competition assay. For that, samples were processed as described above and incubated in a solution of blocking buffer containing 500 mM free GlcNAc previously incubated with CTD110.6 antibody. To assess that CTD110.6 only labeled *O*-beta-linked *N*-acetylglucosamine (27), samples were treated with PNGaseF (New England Biolabs) to remove *N*-glycan chains: a sample of 15 mg of total protein from stimulated ewe 30056 was denatured 10 min at 94°C, digested with PNGase F overnight at 37°C, then inactivated 10 min at 75°C. A western-blot with CTD110.6 as described above was performed.

### 4.8. *In silico* prediction of PTM sites on ewe OS OBPs

These analyses were performed by using the free software at http://www.cbs.dtu.dk/services/NetPhos/, http://www.cbs.dtu.dk/services/YinOYang/, and http://www.cbs.dtu.dk/services/NetNGlyc/, for phosphorylation, *O*-GlcNAcylation and *N*-glycosylation, respectively.

### 4.9. *N*-Glycan chains identification

Dry extracted proteins (300 mg) were resuspended in 500 μl of 50 mM ammonium bicarbonate pH 8.0, reduced by adding 50 μl of 0.1 M DTT for 1 h at 37°C, then alkylated by adding 55 μl of 0.5 M iodoacetamide for 1 h at 37°C in the dark. Proteins were precipitated with trichloroacetic acid (67 μl to reach a final concentration of 10%) 30 min at −20°C. After centrifugation at 13 000 rpm (MiniSpin Eppendorf) for 15 min at 4°C and 3 washes with acetone, the pellet was resuspended with 500 μl of 50 mM ammonium bicarbonate, pH 8.0. Proteins were digested by trypsin (50 μg) at 37°C overnight. After trypsin deactivation at 100°C for 5 min, *N*-glycans were released by an overnight digestion with 5 U of PNGaseF (BioLabs^®^ Inc.) at 37°C. To recover *N*-glycans, the mixture was passed through C18 spin columns (Pierce™) and *N*-glycans were eluted with 5% acetic acid and lyophilized.

Before mass spectrometry analyses, the native *N*-glycans were methylated using a sodium hydroxide permethylation procedure. 100 μl of 50% NaOH solution taken in a clean stoppered glass tube was mixed vigorously with 200 μl of methanol. About 4 mL of anhydrous DMSO was added to this mixture and mixed vigorously by shaking. This process precipitates the carbonates present in the mixture solution as a white fluffy solid. The tube was centrifuged at 13 000 rpm (MiniSpin Eppendorf) for 5 min; this leads to the precipitation of carbonates at the top of the mixture. The precipitated white solid at the top and DMSO in excess were removed carefully while retaining the colorless gel at the bottom of the tube. This procedure was repeated four more times until the white solid precipitation ceased. Further, the resulting NaOH-DMSO gel formed at the bottom of the tube was mixed with 1 ml of anhydrous DMSO and used for the permethylation reaction directly. DMSO (100 μl) was added to the dry sample, sonicated briefly (2 min) in an ultrasonic bath, and allowed to stand at ambient temperature for 15 min. Methylations were carried out by adding 350 μl of the NaOH-DMSO suspension and 50 μl of methyl iodide. After 20 min of incubation, the reaction was stopped by adding 2 mL of cold 5% (v/v) acetic acid, and permethylated N-glycans were recovered by chloroform extraction. The chloroform layer was washed several times with water to remove any impurities and finally dried under a stream of nitrogen.

The permethylated glycans were dissolved in 10 μl of acetonitrile and 1 μl of this solution was mixed with 1 μl of 2,5-dihydroxybenzoic acid (DHB) matrix [10 mg/mL in 50% acetonitrile (v/v)], spotted on a MALDI plate and analyzed on a MALDI-TOF/TOF MS instrument (Axima Resonance, Shimadu Biotech) in reflectron positive mode by delayed extraction using an acceleration mode of 20 kV, a pulse delay of 200 ns and grid voltage of 60%. Between 100 and 150 scans were averaged for each spectrum.

## Data availability statement

The datasets presented in this study can be found in online repositories. The names of the repository/repositories and accession number(s) can be found below: https://www.ncbi.nlm.nih.gov/genbank/, MT921827 and MT921828.

## Ethics statement

The animal study was reviewed and approved by Ethical Committee n° 019 of French “Ministère de l'Enseignement Supérieur, de la Recherche et de l'Innovation” (APAFIS#13067–2018011616242285 v2).

## Author contributions

PNLM, MK, and CLD designed the experiments. MK, CP, and DC set up the male effect protocols. CP performed progesterone assays. CLD performed the nasal mucus sampling, with the help of PC, CP, DC, and MK. PC realized electrophoreses, western-blots, and MALDI-TOF MS. PC, CLD, and PNLM analyzed mass spectrometry data. PNLM cloned the SAL sequences. CLD performed the glycans analysis. All authors contributed to the writing of the manuscript and agree to be accountable for the content of the work.
