# Supplementary material for: Variation of ewe olfactory secretome during a ram effect
Source: Front Vet Sci. 2023 Jan 9;9:1033412. doi: 10.3389/fvets.2022.1033412 (PMC9868937; doi:10.3389/fvets.2022.1033412)

Supplementary Material

1 Supplementary Tables and Figures (order of citation in the text)

Supplementary Table 1. Monitoring of progesterone concentration (ng/mL) in blood of stimulated ewes during the male effect. In yellow, the ewes that responded to the male.

Batch 1			Batch 2		
STIM1	J-1	<0,25	STIM4	J-1	<0,25
STIM1	J0	n. a.	STIM4	J0	<0,25
STIM1	J1	<0,25	STIM4	J1	<0,25
STIM1	J2	<0,25	STIM4	J2	<0,25
STIM1	J3	<0,25	STIM4	J3	<0,25
STIM1	J4	0,3	STIM4	J4	0,8
STIM1	J5	1,2	STIM4	J5	0,5
STIM1	J6	1,5	STIM4	J6	0,6
STIM1	J7	2,3	STIM4	J7	0,8
STIM1	J8	3,2	STIM4	J8	1,3
STIM1	J9	4,4	STIM4	J9	1,1
STIM2	J-1	<0,25	STIM5	J-1	<0,25
STIM2	J0	<0,25	STIM5	J0	<0,25
STIM2	J1	<0,25	STIM5	J1	<0,25
STIM2	J2	0,3	STIM5	J2	<0,25
STIM2	J3	0,9	STIM5	J3	0,4
STIM2	J4	1,4	STIM5	J4	0,9
STIM2	J5	2,1	STIM5	J5	<0,25
STIM2	J6	2,9	STIM5	J6	<0,25
STIM2	J7	3,9	STIM5	J7	<0,25
STIM2	J8	4,7	STIM5	J8	<0,25
STIM2	J9	4,9	STIM5	J9	<0,25
STIM3	J-1	<0,25	STIM6	J-1	<0,25
STIM3	J0	<0,25	STIM6	J0	<0,25
STIM3	J1	0,3	STIM6	J1	0,3
STIM3	J2	<0,25	STIM6	J2	<0,25
STIM3	J3	0,3	STIM6	J3	<0,25
STIM3	J4	0,3	STIM6	J4	<0,25
STIM3	J5	1,1	STIM6	J5	0,3
STIM3	J6	2,5	STIM6	J6	1,4
STIM3	J7	3,5	STIM6	J7	2,0
STIM3	J8	4,3	STIM6	J8	2,9
STIM3	J9	5,1	STIM6	J9	1,4

Supplementary Table 2. Monitoring of progesterone concentration (ng/mL) in blood of control ewes at the same time. In yellow, the "true" control ewes.

Batch 1			Batch 2		
CTRL1	J-1	<0,25	CTRL4	J-1	<0,25
CTRL1	J0	<0,25	CTRL4	J0	<0,25
CTRL1	J1	<0,25	CTRL4	J1	<0,25
CTRL1	J2	<0,25	CTRL4	J2	<0,25
CTRL1	J3	<0,25	CTRL4	J3	0,6
CTRL1	J4	<0,25	CTRL4	J4	<0,25
CTRL1	J5	0,3	CTRL4	J5	0,5
CTRL1	J6	0,3	CTRL4	J6	0,3
CTRL1	J7	<0,25	CTRL4	J7	n. a.
CTRL1	J8	<0,25	CTRL4	J8	<0,25
CTRL1	J9	<0,25	CTRL4	J9	<0,25
CTRL2	J-1	3,2	CTRL5	J-1	<0,25
CTRL2	J0	3,9	CTRL5	J0	<0,25
CTRL2	J1	4,6	CTRL5	J1	<0,25
CTRL2	J2	4,9	CTRL5	J2	<0,25
CTRL2	J3	5,2	CTRL5	J3	<0,25
CTRL2	J4	5,4	CTRL5	J4	<0,25
CTRL2	J5	5,1	CTRL5	J5	<0,25
CTRL2	J6	4,9	CTRL5	J6	<0,25
CTRL2	J7	5,6	CTRL5	J7	<0,25
CTRL2	J8	6,4	CTRL5	J8	<0,25
CTRL2	J9	6,4	CTRL5	J9	<0,25
CTRL3	J-1	1,9	CTRL6	J-1	<0,25
CTRL3	J0	0,8	CTRL6	J0	0,3
CTRL3	J1	1,6	CTRL6	J1	<0,25
CTRL3	J2	0,5	CTRL6	J2	<0,25
CTRL3	J3	0,9	CTRL6	J3	<0,25
CTRL3	J4	1,6	CTRL6	J4	<0,25
CTRL3	J5	1,9	CTRL6	J5	<0,25
CTRL3	J6	1,3	CTRL6	J6	0,6
CTRL3	J7	1,1	CTRL6	J7	0,5
CTRL3	J8	1,1	CTRL6	J8	0,3
CTRL3	J9	1,9	CTRL6	J9	<0,25

Supplementary Figure 1. Two-dimensional electrophoresis of OS extracts along the male effect for stimulated ewes

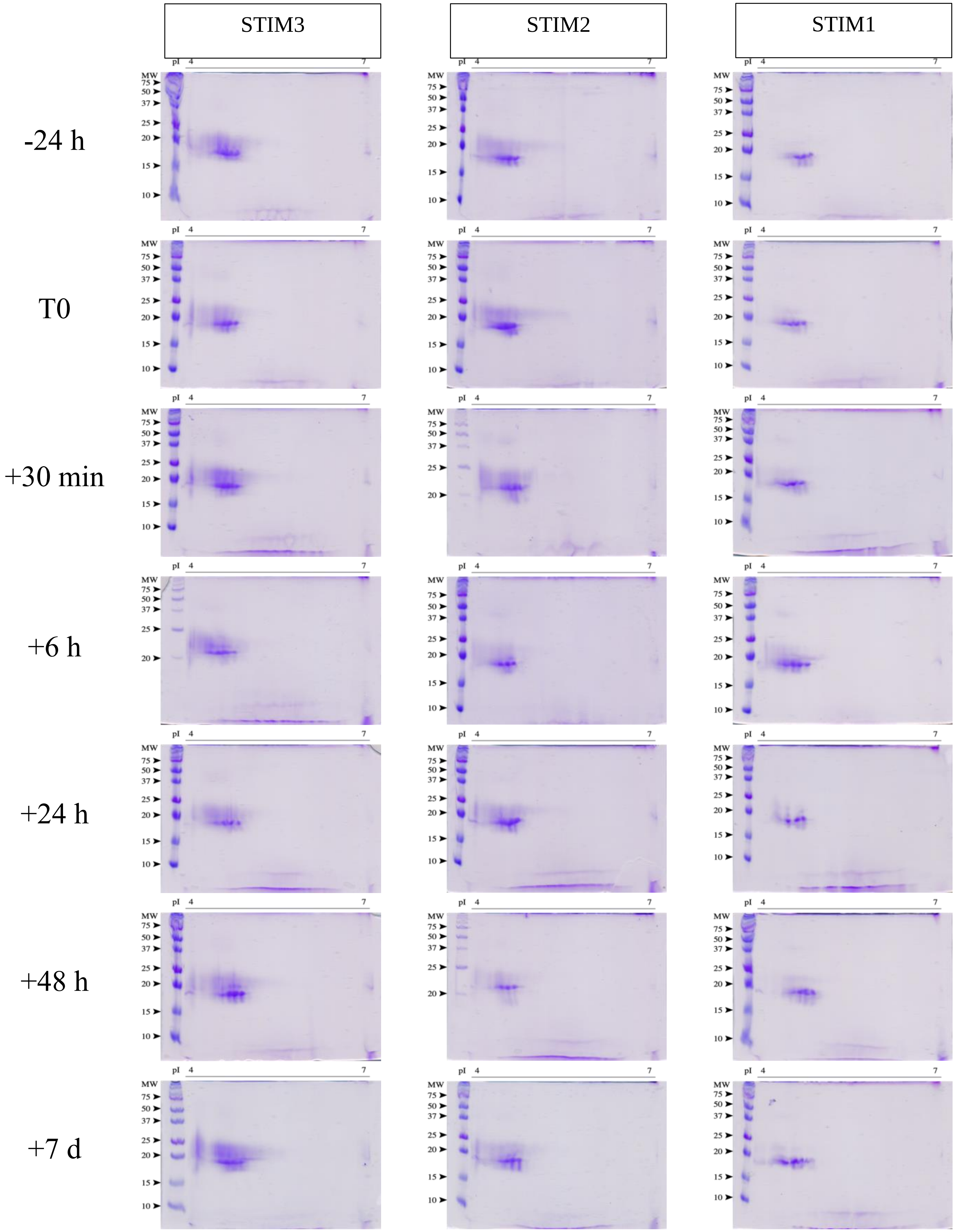

Supplementary Table 3. Odorant-Binding Protein peptides identified by MALDI-TOF MS in 17 kDa band of stimulated ewe STIM1 at each sample time. Corresponding gel in Figure 1.

	Theorical mass (*m/z*)	Peptide position	Modification	-24 h	T0	+30 min	+6 h	+24 h	+48 h	+7 d
				Measured mass (*m/z*)						
Oari-OBP1 W5PHM2	2334.1142	136-155	Cys_CAM:153	2335.8640		2334.8540		2334.9021	2334.9304	
	1191.4946	146-155		1191.4021	1191.4360	1191.4346	1191.4393	1191.4443	1191.4532	1191.4592
	876.4937	138-145		876.9061		876.9252				876.9516
	Sequence coverage %			24.51	6.45	24.51	6.45	19.35	19.35	11.61
Oari-OBP2 *W5PGN0*	2273.0680	121-138		2272.8467	2272.9172	2272.9109		2272.9031	2272.9536	2272.9534
	1799.9115	44-58		1799.6473	1799.7007	1799.6942	1799.7079	1799.7244	1799.7329	1799.7371
	1474.7787	139-151		1474.5352	1474.5891	1474.5819		1474.6034	1474.6047	
	1366.675	29-40		1366.4662	1366.4998	1366.4944	1366.51	1366.5173	1366.5219	1366.5203
	1295.6782	48-58		1295.4648	1295.5135	1295.5164		1295.5215	1295.5215	1295.5265
	920.5200	132-138		920.3638	920.3904	920.3861		920.3998	920.4014	920.4067
	699.4763	114-120		699.3590	699.3850	699.3779	699.3845	699.3799	699.3926	699.4075
	792.425	132-137		792.2956	792.3168	792.3147	792.3208	792.3120	792.3300	792.3290
	Sequence coverage %			41.14	41.14	41.14	25.31	41.14	41.14	41.14
Oari-OBP3 *W5PGW3*	2162.9182	136-154	Cys_PAM: 151	2162.7463	2162.8284	2162.8228		2162.8433	2162.854	2162.8477
	1925.9814	89-106		1925.7269						
	1394.7022	9-22		1394.474	1394.5054	1394.5045	1394.515	1394.5171	1394.5262	1394.5291
	Sequence coverage %			33.33	21.56	21.56	9.15	21.56	21.56	21.56
Oari-OBP4 *W5PHS2*	1433.7318	29-40	Cys_CAM: 37	1433.5159	1433.5526	1433.5486	1433.5211			
	1099.5605	63-71	Cys_PAM: 63							1099.4581
	840.4937	29-36		840.3596		840.3658				
	792.392	113-119	Cys_PAM: 117	792.2956	792.3168	792.3147	792.3208	792.3120	792.3300	792.3290
	Sequence coverage %			17.30	12.17	17.30	12.17	4.48	4.48	10.25

Supplementary Table 4. Odorant-Binding Protein peptides identified by MALDI-TOF MS in 20 kDa band of stimulated ewe STIM1 at each sample time. Corresponding gel in Figure 1.

	Theorical mass (*m/z*)	Peptide position	Modification	-24 h	T0	+30 min	+6 h	+24 h	+48 h	+7 d
				Measured mass (*m/z*)						
Oari-OBP1 *W5PHM2*	2495.3391	89-111							2495.7942	
	2334.1142	136-155	Cys_CAM:153						2334.7073	
	1191.4946	146-155					1191.3422			
	876.4937	138-145					876.8616	876.8731		876.8436
	Sequence coverage %			0	0	0	12.25	5.16	27.74	5.16
Oari-OBP2 *W5PGN0*	1799.9115	44-58		1799.5225		1799.5841	1799.5670	1799.6028	1799.5552	1799.542
	2273.0680	121-138		2272.8467		2272.9109	2272.7668	2272.9031	2272.9534	2272.5446
	1602.8737	138-151					1602.5671			
	1366.675	29-40		1366.3523	1366.3826	1366.3993	1366.3877	1366.4020	1366.3717	1366.3660
	1371.5658	121-131							1370.9965	1370.9049
	920.52	132-138		920.3036			920.3106	920.3451		
	792.425	132-137		792.2295		792.2466	792.2504	792.2695		
	Sequence coverage %			29.11	7.59	29.11	37.97	29.11	29.11	29.11
Oari-OBP3 W5PGW3	2162.9182	136-154	Cys_PAM: 151	2162.5857	2162.8245	2162.8093		2162.7664	2162.8638	2462.8633
	Sequence coverage %			5.88	5.88	5.88	0	5.88	5.88	5.88
Oari-SAL	1295.7219	93-102					1295.4208			
	1721.8857	17-31		1721.5260			1721.5667	1721.5874	1721.5490	1721.5507
	1519.8478	142-154					1519.5502		1519.5042	1519.5271
	992.5523	95-102					992.3285		992.3405	992.3121
	Sequence coverage %			14.97	0	0	28.74	14.97	22.75	22.75

Supplementary Table 5. Odorant-Binding Protein peptides identified by MALDI-TOF MS in 17 kDa band of stimulated ewe STIM2 at each sample time. Corresponding gel in Figure 1.

	Theorical mass (*m/z*)	Peptide position	Modification	-24 h	T0	+30 min	+6 h	+24 h	+48 h	+7 d
				Measured mass (*m/z*)						
Oari-OBP1 *W5PHM2*	2334.1142	136-155	Cys_CAM:153	2334.8656		2334.8599				2334.8818
	1191.4946	146-155		1191.4174	1191.4094	1191.4156	1191.4133	1191.4161	1191.4260	1191.4266
	876.4937	138-145		876.9111	876.9124	876.9135	876.9139	876.9129	876.9167	876.9257
	Sequence coverage %			24.51	11.61	24.51	11.61	11.61	11,61	24.51
Oari-OBP2 *W5PGN0*	2273.1142	121-138		2272.8875	2272.8774	2272.8979	2272.8931	2272.8989	2272.8975	2272.8936
	1799.9115	44-58		1799.6846	1799.6735	1799.6770	1799.6824	1799.6838	1799.6937	1799.6910
	1474.7787	139-151		1474.5721	1474.5570	1474.5734	1474.5687	1474.5724	1474.5757	1474.5718
	1366.675	29-40		1366.4829	1366.4819	1366.4845	1366.4918	1366.4944	1366.4896	1366.4867
	1295.6782	48-58		1295.4939	1295.4811	1295.5049	1295.4897	1295.4957		1295.5085
	920.52	132-138		920.3763	920.3684	920.3792	920.3758	920.3804	920.3858	920.3829
	792.425	132-137		792.2995	792.2939	792.3029	792.3003	792.2791	792.3116	792.3074
	Sequence coverage %			29.74	29.74	29.74	29.74	29.74	29.74	29.74
Oari-OBP3 *W5PGW3*	2162.9182	136-154	Cys_PAM:151	2162.8027	2162.8042	2162.8208	2162.8135	2162.8084		2162.8088
	1925.9814	89-106							1925.7061	
	Sequence coverage %			12.40	12.40	12.40	12.40	12.40	11.76	12.40
Oari-OBP4 *W5PHS2*	1967.9821	12-28		1967.7393	1967.7410	1967.1405	1967.7455	1967.7526	1967.7488	1967.7439
	1051.5167	131-138		1051.3676	1051.3536		1051.3643	1051.3746	1051.3752	
	792.3920	113-119	Cys_PAM: 117	792.2995	792.2989	792.3029	792.3050	792.2791	792.3116	792.3074
	747.3784	12-17		747.2626	747.2634	747.2726	747.2683	747.2791	747.2711	747.2707
	Sequence coverage %			19.16	19.16	14.37	19.16	19.16	19.16	14.37

Supplementary Table 6. Odorant-Binding Protein peptides identified by MALDI-TOF MS in 20 kDa band of stimulated ewe STIM2 at each sample time. Corresponding gel in Figure 1.

	Theorical mass (*m/z*)	Peptide position	Modification	-24 h	T0	+30 min	+6 h	+24 h	+48 h	+7 d
				Measured mass (*m/z*)						
Oari-OBP1 *W5PHM2*	Sequence coverage %			0	0	0	0	0	0	0
Oari-OBP2 *W5PGN0*	2598.2583	18-40		2598.8340			2598.7468		2598.8538	2598.9556
	2273.0680	121-138		2272.7610				2272.7661	2272.7454	2272.8459
	1799.9115	44-58		1799.5546	1799.6152	1799.6400	1799.5378	1799.5061	1799.5344	1799.6241
	1366.675	29-40		1366.3801	1366.4104	1366.4203	1366.3835	1366.3602	1366.3572	1366.4196
	792.425	132-137		792.2386				792.2504	792.2243	792.2618
	Sequence coverage %			36.07	17.08	17.08	23.95	27.54	36.07	36.07
Oari-OBP3 *W5PGW3*	2162.9182	136-154	Cys_PAM: 151	2162.6770					2162.6533	2162.7256
	Sequence coverage %			13.72	0	0	0	0	13.72	13.72
Oari-OBP4 *W5PHS2*	1184.6244	63-72		1184.3573	1184.4005	1184.4148	1184.3486	1184.5013	1184.3422	1184.3879
	792.3920	113-119	Cys_PAM:117	792.2474				792.2504	792.2243	792.2618
	747.3784	12-17		747.2154						
	Sequence coverage %			14.74	6.53	6.53	6.53	10.17	10.17	10.17
Oari-SAL	1721.8857	17-31						1721.5775		1721.6123
	1295.7219	95-102							1295.3693	
	992.5523	95-102		992.3243	992.3594					
	Sequence coverage %			4.79	4.79	0	0	8.98	4.79	8.98

Supplementary Table 7. Odorant-Binding Protein peptides identified by MALDI-TOF MS in 17 kDa band of stimulated ewe STIM3 at each sample time. Corresponding gel in Figure 1.

	Theorical mass (*m/z*)	Peptide position	Modification	-24 h	T0	+30 min	+6 h	+24 h	+48 h	+7 d
				Measured mass (*m/z*)						
Oari-OBP1 *W5PHM2*	1191.4946	146-155	Cys_CAM:153	1191.4656	1191.4609	1191.4578	1191.4404	1191.4227	1191.4357	1191.4209
	876.4937	138-145				876.9497	876.9314	876.9233		
	Sequence coverage %			7.09	7.09	12.25	12.25	12.25	7.09	7.09
Oari-OBP2 *W5PGN0*	2273.068	121-138		2272.9646	2272.9521	2272.9822	2272.9448	2272.9619	2272.9246	2272.8799
	1799.9115	44-58		1799.7236	1799.7477	1799.7524	1799.7228	1799.7181	1799.7097	1799.6588
	1474.7787	139-151			1474.6184	1474.6239	1474.6058	1474.5891	1474.572	
	1366.675	29-40		1366.5345	1366.5408	1366.5409	1366.5134	1366.5103	1366.5112	1366.4778
	1295.6782	48-58		1295.5417	1295.5341		1295.5221	1295.5072	1295.5056	1295.4822
	920.52	132-138		920.4209	920.411	920.4122	920.3956	920.3896	920.3915	920.3778
	792.425	132-137		792.3358	792.3314	792.3327	792.3173	792.3132	792.3187	792.3021
	Sequence coverage %			29.11	37.34	29.11	37.34	37.34	37.34	29.11
Oari-OBP3 *W5PGW3*	2162.9182	136-154	Cys_PAM: 151	2162.8533	2162.8789	2162.8894	2162.8767	2162.8608	2162.8115	2162.9507
	1925.9814	89-106			1925.7755	1925.7648	1925.7356	1925.736	1925.7284	
	1394.7022	9-22							1394.5129	1394.4888
	Sequence coverage %			13.72	24.18	24.18	24.18	24.18	33.33	21.56
Oari-OBP4 *W5PHS2*	1301.6008	120-130				1301.4603	1301.4648			
	1051.5167	131-138			1051.4000	1051.4026	1051.3851	1051.3761	1051.3794	
	792.3920	113-119		792.3021	792.3358	792.3314	792.3327	792.3173	792.3132	792.3187
	Sequence coverage %			4.48	9.61	16.66	16.66	9.61	9.61	4.48

Supplementary Table 8. Odorant-Binding Protein peptides identified by MALDI-TOF MS in 20 kDa band of stimulated ewe STIM3 at each sample time. Corresponding gel in Figure 1.

	Theorical mass (*m/z*)	Peptide position	Modification	-24 h	T0	+30 min	+6 h	+24 h	+48 h	+7 d
				Measured mass (*m/z*)						
Oari-OBP1 W5PHM2	2495.3391	89-111					2495.8833			
	2334.1142	136-155	Cys_CAM:153	2334.7791						
	876.4937	138-145		876.8671						
	Sequence coverage %			13.54	0	0	14.19	0	0	0
Oari-OBP2 *W5PGN0*	2273.0680	121-138		2272.9026	2272.8286	2272.8127	2272.7986	2272.8071	2272.7603	
	1799.9115	44-58		1799.6680	1799.6173	1799.6268	1799.8793	1799.6162	1799.549	1799.5614
	1371.5658	121-131			1371.4082	1370.9961	1371.9452	1370.9839		1370.9911
	1366.6750	29-40		1366.4481	1366.411	1366.417	1366.6144	1366.3955	1366.3724	1366.3744
	792.4250	132-137			792.2620				792.2333	
	Sequence coverage %			29.11	29.11	29.11	29.11	29.11	29.11	24.05
Oari-OBP3 *W5PGW3*	2162.9182	136-154	Cys_PAM:151	2162.8835	2162.7024	2162.7207	2162.7371		2162.6423	2162.6753
	1978.0756	69-85							1978.2738	
	Sequence coverage %			12.41	12.41	12.41	12.41	0	23.53	12.41
Oari-OBP4 *W5PHS2*	792.3920	113-119	Cys_PAM:117		792.2620				792.2333	
	1184.6244	63-72		1184.4117	1184.3823	1184.3937	1184.3751	1184.3618		1184.3523
	Sequence coverage %			6.41	10.89	6.41	6.41	6.41	4.48	6.41
Oari-SAL	1721.8857	17-31		1721.6495	1721.6008	1721.5927	1721.5942	1721.5792	1721.5558	1721.5466
	1519.8478	142-154		1519.6158			1519.5676		1519.5238	
	992.5523	95-102		992.3637	992.3424			992.3289	992.3177	992.3238
	959.5156	147-154							959.2902	959.3065
	Sequence coverage %			20.35	13.77	8.98	15.56	13.77	20.35	18.56

Supplementary Table 9. Odorant-Binding Protein peptides identified by MALDI-TOF MS in 17 kDa band of control ewe CTRL1 at each sample time. Corresponding gel in Figure 2.

	Theorical mass (*m/z*)	Peptide position	Modification	-24 h	T0	+30 min	+6 h	+24 h	+48 h	+7 d
				Measured mass (*m/z*)						
Oari-OBP1	2334.1142	136-155	Cys_CAM: 153		2334.8301	2334.8371	2334.8691	2334.8306	2334.8326	2334.8542
W5PHM2	876.4937	138-145		876.9471	876.9142					876.9229
	Sequence coverage %			5.16	13.54	13.54	13.54	13.54	13.54	13.54
Oari-OBP2										
	2273.0680	121-138		2272.9932	2272.8845	2272.9258	2272.9316		2272.9001	2272.9143
	1799.9115	44-58		1799.7578	1799.6820	1799.7109	1799.6969	1799.6814	1799.7008	1799.707
	1474.7787	139-151		1474.6282	1474.5714	1474.5906	1474.5951	1474.5713	1474.5741	1474.5764
	1366.6750	29-40		1366.5651	1366.4957	1366.5226	1366.5007	1366.4902	1366.5002	1366.5204
	1295.6782	48-58		1295.5409	1295.4918		1295.5177	1295.5027	1295.5093	1295.4913
	920.5200	132-138		920.4133	920.3744	920.3819	920.3896	920.3815	920.381	920.3799
	792.4250	132-137		792.3338	792.2997	792.3069	792.3178	792.3111	792.3072	792.3055
	Sequence coverage %			36.70	36.70	36.70	36.70	29.74	36.70	36.70
Oari-OBP3	2162.9182	136-154	Cys_PAM: 151	2162.8926	2162.8269	2162.8611	2162.8496	2162.8284	2162.7903	2162.8293
W5PGW3	1925.9814	89-106		1925.7968	1925.6904	1925.7228	1925.7303		1925.7028	1925.7240
	1394.7022	9-22		1394.5388	1394.4757	1394.4908	1394.5067	1394.4946	1394.4961	1394.4911
	Sequence coverage %			33.33	36.70	36.70	36.70	21.56	36.70	36.70
Oari-OBP4	1051.5167	131-138		1051.4033	1051.3585	1051.3661	1051.3845	1051.3704	1051.3640	1051.3629
	792.3920	113-119	Cys_PAM:117	792.3338	792.2997	792.3069	792.3178	792.3111	792.3072	792.3055
	747.3784	12-17		747.2714	747.2816	747.2689	747.2783		747.2731	747.2718
	Sequence coverage %			13.46	13.46	13.46	13.46	9.61	13.46	13.46

Supplementary Table 10. Odorant-Binding Protein peptides identified by MALDI-TOF MS in 20 kDa band of control ewe CTRL1 at each sample time. Corresponding gel in Figure 2.

	Theorical mass (*m/z*)	Peptide position	Modification	-24 h	T0	+30 min	+6 h	+24 h	+48 h	+7 d
				Measured mass (*m/z*)						
Oari-OBP1 *W5PHM2*	876.4937	138-145			876.8206		876.8359			876.8489
		Sequence coverage %		0	5.16	0	5.16	0	0	5.16
Oari-OBP2 *W5PGN0*	1799.9115	44-58		1799.5896	1799.5239	1799.3696	1799.3380	1799.5532	1799.5585	1799.3547
	1366.675	29-40		1366.3916	1366.348	1366.3734	1366.3632	1366.3704	1366.3796	1366.363
	792.425	132-137		792.2565	792.2279	792.2350			792.2474	
	2273.0680	121-138		2272.8291		2272.7417		2272.7332	2272.7493	
		Sequence coverage %		29.11	20.88	29.11	17.08	29.11	29.11	17.08
Oari-OBP3 W5PGW3	2162.9182	136-154	Cys_PAM: 151	2162.6865	2162.6428	2162.6611		2162.6804	2162.634	2162.6421
	1978.0756	69-85		1978.6966	1978.6740			1978.6477	1978.6914	1978.6780
		Sequence coverage %		23.53	23.53	12.41	0	23.53	23.53	23.53
Oari-OBP4 *W5PHS2*	1184.6244	63-72		1184.3514	1184.3250	1184.3438		1184.3461	1184.3724	
	792.3920	113-119	Cys_CAM: 117	792.2565	792.2279	792.2350			792.2474	
		Sequence coverage %		10.89	7.69	10.89	0	6.41	10.89	0
Oari-SAL	1721.8857	17-31		1721.5833		1721.5502	1721.5341	1721.5417	1721.5640	1721.3547
	1519.8478	142-154		1519.5388	1519.5018	1519.512		1519.5065	1519.5293	1519.5171
	959.5156	147-154		959.3022	959.2714		959.2808	959.2911	959.3045	959.2919
	992.5523	95-102		992.3200	992.2917	992.3049	992.3125	992.3083	992.3228	
		Sequence coverage %		21.55	12.57	21.55	18.56	21.55	21.55	16.76

Supplementary Table 11. Odorant-Binding Protein's peptides identified by MALDI-TOF MS in 17 kDa band of control ewe CTRL2 at each sample time. Corresponding gel in Figure 2.

	Theorical mass (*m/z*)	Peptide position	Modification	-24 h	T0	+30 min	+6 h	+24 h	+48 h	+7 d
				Measured mass (*m/z*)						
Oari-OBP1 *W5PHM2*	2334.1142	136-155	Cys_CAM: 153	2334.0876	2334.5786	2334.3480		2334.4680		
	Sequence coverage %			13.54	13.54	13.54	0	13.54	0	0
Oari-OBP2 *W5PGN0*	2273.0680	121-138		2272.8867	2272.8760	2272.9231	2272.9302	2272.9138	2272.9243	2272.9131
	1694.8860	29-43							1694.7229	1694.6542
	1366.6750	29-40		1366.4863		1366.5411			1366.4945	
	1371.5658	121-131			1371.0881	1371.4660	1371.3080	1371.5680	1371.3780	1371.4970
	Sequence coverage %			18.98	11.39	18.98	18.98	18.98	20.88	20.88
Oari-OBP3 *W5PGW3*	2162.9182	136-154	Cys_PAM: 151	2162.7817	2162.7808	2162.8137	2162.8335	2162.8101	2162.8267	2162.8174
	1925.9814	89-106		1925.6736		1925.6997	1925.7047	1925.6897	1925.7001	1925.6936
	1500.7216	118-129		1500.5576		1500.5869	1500.5927	1500.5691	1500.5847	
	Sequence coverage %			32.02	12.41	32.02	32.02	32.02	32.02	24.18
Oari-OBP4 *W5PHS2*	1051.5167	131-138		1051.3608		1051.3667	1051.3634	1051.3602	1051.3610	1051.3666
	1730.8795	59-72	Cys_PAM: 63		1730.6716					
	1301.6008	120-130							1301.4209	
	1241.6459	63-72	Cys_CAM: 63	1241.4633		1241.4655	1241.4683	1241.4590	1241.4635	1241.4672
	1184.6244	63-72			1184.4653	1184.4633	1184.4689	1184.4551	1184.4524	1184.4625
	840.4937	29-36		840.3693		840.3683	840.3672	840.3644	840.3654	840.3691
	747.3784	12-17		747.2623	747.2870	747.2664	747.2634	747.2623	747.2625	747.2656
	Sequence coverage %			20.51	19.23	20.51	20.51	20.51	27.56	20.51
Oari-SAL	1721.8857	17-31		1721.6682	1721.6995	1721.6827	1721.7003	1721.6691	1721.6621	1721.6532
	1329.7121	147-157		1329.4437		1329.4634	1329.4673	1329.4423	1329.4407	1329.4409
	1051.5101	155-162	Cys_CAM: 158	1051.3608		1051.3667	1051.3634	1051.3602	1051.361	1051.3666
	Sequence coverage %			15.56	8.98	15.56	15.56	15.56	15.56	15.56

Supplementary Table 12. Odorant-Binding Protein peptide identified by MALDI-TOF MS in 20 kDa band of control ewe CTRL2 at each sample time. Corresponding gel in Figure 2.

	Theorical mass (*m/z*)	Peptide position	Modification	-24 h	T0	+30 min	+6 h	+24 h	+48 h	+7 d
				Measured mass (*m/z*)						
Oari-OBP1 *W5PHM2*	2334.1142	136-155	Cys_CAM: 153		2334.7144	2334.6245				
	Sequence coverage %			0	13.54	13.54	0	0	0	0
Oari-OBP2 W5PGN0	2273.0680	121-138		2272.8193	2272.7656					
	1371.5658	121-131		1370.9459	1370.8690		1370.9166	1370.9016	1370.9297	1370.9214
	Sequence coverage %			12.02	12.02	0	6.96	6.96	6.96	6.96
Oari-OBP3 W5PGW3	2162.9182	136-154	Cys_PAM:51	2162.7407						2162.6287
	Sequence coverage %			12.41	0	0	0	0	0	12.41
Oari-OBP4 *W5PHS2*	2143.9674	42-58	Cys_PAM: 44. 48		2143.7039					
	1184.6244	63-72		1184.3522	1184.5115	1184.3922	1184.3580	1184.3618	1184.3462	
	1051.5167	131-138			1051.2788					
	Sequence coverage %			6.41	22.43	6.41	6.41	6.41	6.41	0
Oari-SAL	3136.5619	32-58	MSO: 40							3136.401
	1519.8478	142-154						1519.5353	1519.4938	1519.6287
	992.5523	95-102		992.3154		992.3554	992.3228	992.3251	992.3308	992.2933
	1721.8857	147-154					1721.5262	1721.5459	1721.5426	
	Sequence coverage %			4.79	0	4.79	13.77	12.57	12.57	28.74

Supplementary Table 13. Odorant-Binding Protein peptides identified by MALDI-TOF MS in 17 kDa band of control ewe CTRL3 at each sampling time. Corresponding gel in Figure 2.

	Theorical mass (*m/z*)	Peptide position	Modification	-24 h	T0	+30 min	+6 h	+24 h	+48 h	+7 d
				Measured mass (*m/z*)						
Oari-OBP1 *W5PHM2*	2048.9705	138-155	cys_CAM: 153				2048.7029		2048.7046	
	Sequence coverage %			0	0	0	11.61	0	11.61	0
Oari-OBP2 *W5PGN0*	2273.0680	121-138		2272.9221	2272.9321	2272.9246	2272.9258	2272.9377	2272.9277	2272.9421
	1694.8860	29-43		1694.6021			1694.5983	1694.6554	1694.6171	1694.6210
	1371.5658	121-131		1371.0671	1371.1157	1371.0813	1371.0681	1371.0747	1371.0647	1371.0826
	Sequence coverage %			20.88	18.35	18.35	20.88	20.88	20.88	20.88
Oari-OBP3 *W5PGW3*	2162.9182	136-154	Cys_PAM: 151	2162.8267	2162.8284	2162.8315	2162.8196	2162.8577	2162.8298	2162.8433
	1925.9814	89-106		1925.7019	1925.7045	1925.7054	1925.6974	1925.7363	1925.7054	1925.7145
	Sequence coverage %			24.18	24.18	24.18	24.18	24.18	24.18	24.18
Oari-OBP4 *W5PHS2*	1301.6008	120-130						1301.4468		1301.4409
	1241.6459	63-72	Cys_CAM: 63	1241.4766	1241.4709	1241.4758	1241.4802	1241.4832	1241.4808	1241.4874
	1184.6244	63-72		1184.4641		1184.4656	1184.4691	1184.4789	1184.4812	1184.4738
	1085.5448	63-71	Cys_CAM: 63		1085.4011					
	840.4937	29-36		840.3727	840.3808	840.3758	840.3712	840.3814	840.3771	840.3805
	747.3784	12-17		747.2692	747.2672	747.2707	747.2684	747.277	747.2725	747.2772
	Sequence coverage %			15.38	15.38	15.38	15.38	22.43	15.38	22.43
Oari-SAL	1329.7121	147-157		1329.4496	1329.4587	1329.4539	1329.4470	1329.4630	1329.4565	1329.4602
	1079.5051	158-167	Cys_CAM: 158		1079.3887		1079.3854	1079.3982		
	1051.5101	155-162	Cys_CAM: 158	1051.3690	1051.3715	1051.3732	1051.3674	1051.3799	1051.3757	1051.3809
	Sequence coverage %			6.58	12.57	11.37	12.57	12.57	11.37	11.37
Oari-VEG1 W5NUS5	2024.8872	119-135				2024.6901				2024.7455
	986.6033	85-92		986.4670	986.4621	986.4641	986.4630		986.4677	986.4728
	Sequence coverage %			5.09	5.09	15.92	5.09	0	5.09	15.92

Supplementary Table 14. Odorant-Binding Protein peptides identified by MALDI-TOF MS in 20 kDa band of control ewe CTRL3 at each sampling time. Corresponding gel in Figure 2.

	Theoretical mass (*m/z*)	Peptide position	Modification	-24 h	T0	+30 min	+6 h	+24 h	+48 h	+7 d
				Measured mass (*m/z*)						
Oari-OBP1 *W5PHM2*	2334.1142	136-155	Cys_CAM: 153				2334.7209			
	1461.7696	29-40					1461.4358		1461.4511	
	Sequence coverage %			0	0	0	21.29	0	7.74	0
Oari-OBP2 *W5PGN0*	2273.0680	121-138		2272 .6965			2272.7771		2272.8723	2272.7961
	1371.5658	121-131		1370.9133	1370.9822	1370.9539	1370.9333	1370.9524	1370.9587	1370.9896
	Sequence coverage %			11.39	6.96	6.96	11.39	6.96	11.39	11.39
Oari-OBP3 *W5PGW3*	1978.0756	69-85						1978.69812		
	2162.9182	136-154	Cys_PAM:51					2162.7188	2162.7139	2162.7961
	Sequence coverage %			0	0	0	0	24.18	13.07	13.07
Oari-OBP4 *W5PHS2*	1184.6244	63-72	Cys_PAM:44. 48	1184.3734	1184.3629	1184.3617	1184.3693	1184.3834	1184.3826	1184.3790
	Sequence coverage %			6.41	6.41	6.41	6.41	6.41	6.41	6.41
Oari-SAL	1721.8857	17-31		1721.5618	1721.5479	1721.5687		1721.5936		1721.5996
	1519.8478	142-154				1519.5278		1519.5670		1519.5767
	992.5523	95-102		992.3347	992.3353	992.3134	992.3151	992.4636	992.3379	
	Sequence coverage %			13.77	13.77	21.55	4.79	21.55	4.79	16.76

Supplementary Figure 2. Molecular cloning of SAL-like sequences. A – Oari-SAL sequences from Ensembl database. **B** – Alignment of protein sequences translated from the six different clones obtained by RACE-PCR from CTRL1 ewe in SA nasal mucosa.

A –

OariSAL1

```
>tr|W5P8W4|W5P8W4_SHEEP Major allergen Equ c 1-like OS=Ovis aries OX=9940 GN=LOC101109939 PE=3 SV=1
MKLLLLCLGLTLVRAQEGNSDVVRSNFDIPKIAGEWYSILLASDNREKIEENGSMRFFVE
HISLLENSSLFIKMHTKVNGVCTELPLTCDSTGEDGVYTVSYDGNNKFRILQVNYSHHII
FYLENFSDSFQLLELYAREPDTSPELKNEFVEICQKYGIVKENVIDLTRVDRCFQARGNG
VA
```

OariSAL2

```
>tr|W5P8Y1|W5P8Y1_SHEEP Major allergen Equ c 1-like OS=Ovis aries OX=9940 GN=LOC101110208 PE=3 SV=1
MKLLLLCLGLTLVCAQEGTSDVVRSNFDIPKIAGEWYSILLASDHREKIEENGSMRVFVE
HIDVLENSSLSFKFHTKVNGVCTELPLVSDSTGEDGVYTISYDGNNTFRILQVNYSRHII
FYLENFSDSYQLLELYAREPDTSPELKNEFVEICQKYGIVKENIIDLTRVDRCFQARGNG
VA
```

B –

```
CLUSTAL O(1.2.4) multiple sequence alignment


VIRT-61482:5'3'      MKLLLLCLGLTLVCAQEGTSDVVRSNFDIPKIAGEWYSILLASDHREKIEENGSMRVFVE   60
VIRT-41714:5'3'      MKLLLLCLGLTLVRAQEGNSDVVRSNFDIPKITGEWFSILLASDNREKIEENGSMRFFVE   60
VIRT-37845:5'3'      MKLLLLCLGLTLVCAQEGTSDVVRSNFDIPKITGEWFSILLASDNREKIEENGSMRFFVE   60
VIRT-44555:5'3'      MKLLLLCLGLTLVRAQEGNSDVVRSNFDIPKITGEWFSILLASDSREKIEENGSMRFFVE   60
VIRT-33326:3'5'      MKLLLLCLGLTLVRAQEGNSDVVRSNFDIPKITGEWFSILLASDNREKIEENGSMRFFVE   60
VIRT-49626:3'5'      MKLLLLCLGLTLVRAQEGNSDVVRSNFDIPKITGEWFSILLASDNREKIEENGSMRFFVE   60
                     ************* ****.*************:***:******* ***********.***

VIRT-61482:5'3'      HIDVLENSSLSFKFHTKVNGVCTELPLVSDSTGEDGIYTISYDGNNKFRILQVNYSRHII   120
```

```
VIRT-41714:5'3'      HISLLENSSLFIKMHTKVNGVCTELPLTCDSTGEDGVYTVSYDGNNKFRILQVNYSRHII   120
VIRT-37845:5'3'      HISLLENSSLFIKMHTKVNGVCTELPLTCDSTGEDGVYTVSYDGNNTFRILQVNYSHHII   120
VIRT-44555:5'3'      HISLLENSSLFIKMHTKVNGVCTELPLTCDSTGEDGVYTVSYDGNNTFRILQVNYSHHII   120
VIRT-33326:3'5'      HISLLENSSLFIKMHTKVNGVCTELPLTCDSTGEDGVYTVSYDGNNTFRILQVNYSHHII   120
VIRT-49626:3'5'      HISLLENSSLFIKMHTKVNGVCTELPLTCDSTGEDGVYTVSYDGNNTFRILQVNYSHHII   120
                     **.:****** :*:*************..*******:**:******.*********:***

VIRT-61482:5'3'      FYLENFSDSYQLLELYAREPDTSPELKNEFVEICQKYGIVKENVIDLTRVDRCFQARGNG   180
VIRT-41714:5'3'      FYLENFSDSFQLLELYAREPDTSPELKNEFVEICQKYGIVKENVTDLTRVDRCFQARGNG   180
VIRT-37845:5'3'      FYLENFSDSFQLLELYAREPDTSPELKNEFVEICQKYGIVKENVIDLTRVDRCFQARGNG   180
VIRT-44555:5'3'      FYLENFSDSFQLLELYAREPDTSPELKNEFVEICQKYGIVKENVIDLTRVDRCFQARGNG   180
VIRT-33326:3'5'      FYLENFSDSFQLLELYAREPDTSPELKNEFVEICQKYGIVKENVVDLTRVDRCFQARGNG   180
VIRT-49626:3'5'      FYLENFSDSFQLLELYAREPDTSPELKNEFVEICQKYGIVKENVIDLTRVDRCFQARGNG   180
                     *********:********************************** ***************

VIRT-61482:5'3'      VA        182
VIRT-41714:5'3'      VA        182
VIRT-37845:5'3'      VA        182
VIRT-44555:5'3'      VA        182
VIRT-33326:3'5'      VA        182
VIRT-49626:3'5'      VA        182
                     **
```

Supplementary Figure 3. Predicted sites of post-translational modifications on Oari-OBPs, Oari-SALs, and Oari-VEG sequences. Phosphorylation in green, *O*-GlcNAcylation in red, and *N*-glycosylation in purple (consensus pattern NXS/T in bold).

>Oari-OBP1_W5PHM2
QEAPAELDPSQITGDWRSILTAADNKEKIEEEGPLRTYVRRLECIDSCSSLSIKFYAKFPKQCTFLNIVAEREGDVYQVGYMGSNSFKLILVSENSLAVYGENFDGVKVT
KVTQLLAKGDGTTEEETQQYEELNKERGIPPEHVKDLTQTDNCPQ

>Oari-OBP2-QEY02201
QEIPAEPHHSEISGEWRTHYIASSNTEKTGENGPFNVYLRSIKFNDKGDSLVFHFFVKNNGACTESSVSGRRIANNVYVAEYAGANEFHFILVSDDGLIVNSENVDEAG**N**
RTRLVGLLGKEDEVDDHDLERFLEEVRKLGIPEENIVDFTKGDGCQAQ

>Oari-OBP3_W5PGW3
EEEGGSSRSFTGINSLNSAAGRGSCPGTNQGLEPLLQSFLETGKASAIFTIFVSSNGECVKKQVTGEKEKISVYHITYAGQNKVKILRLSLDTIIGSIHN
VDEDGKETELVGILGKRDQISDIDYEKFKKEASDRGIPEENIV**NFT**DNDDCPAE

>Oari-OBP4_QEY02202
QETPAEIDPSKVTGEWRTIYSAADNKEKIVEGGPLRCYNRKIECTDDCEHLSISFYVKFDGRCQFFSGVLKRQEGGVYFIEFEGANYLQIIHVSDNILVLYFENDDGQKI
TKLTEGCAKGTSFTQEEFQKYQQLNTERGIPNENIEHVIETDDCPP

>Oari-SAL1_W5P8W4
QEGNSDVVRSNFDIPKIAGEWYSILLASDNREKIEE**NGS**MRFFVEHISLLE**NSS**LFIKMHTKVNGVCTELPLTCDSTGEDGVYTVSYDGNNKFRILQV**NYS**HHIIFYLE**N**
FSDSFQLLELYAREPDTSPELKNEFVEICQKYGIVKENVIDLTRVDRCFQARGNGVA

>oari-SAL2_W5P8Y1
QEGTSDVVRSNFDIPKIAGEWYSILLASDHREKIEE**NGS**MRVFVEHIDVLE**NSS**LSFKFHTKVNGVCTELPLVSDSTGEDGVYTISYDG**NNT**FRILQV**NYS**RHIIFYLE**N**
FSDSYQLLELYAREPDTSPELKNEFVEICQKYGIVKENIIDLTRVDRCFQARGNGVA

>Oari-VEG1_W5NUS5
QDALVLDSWTEDVSGKWYLKAVTTDQDVPGKNQESVTAMTFSVLEGGDLEAKVTLRVDGQCQETGLVLEQTNDPGRYTAYGGKREVFILPLRAQDHFILYCEGELGGRQI
RVARLLGRNPENSPEAWEEFTEFAKAKKLNLKIFRPLQSETCSPRGN

Supplementary Figure 4. Control of CTD110.6 antibody specificity on proteins extracted from olfactory secretome of stimulated ewe STIM1. A - Film exposed to the membrane incubated with a solution of CTD110.6 (1/5,000 dilution) and 1M free GlcNAc for competition assay. **B** – Western-blot with anti-*O*-GlcNAc antibody (CTD110.6) after PNGase F treatment.
SDS-PAGE of 15 µg of proteins per well, 500 ng of *O*-GlcNAcylated BSA (Carbosynth) as positive control.

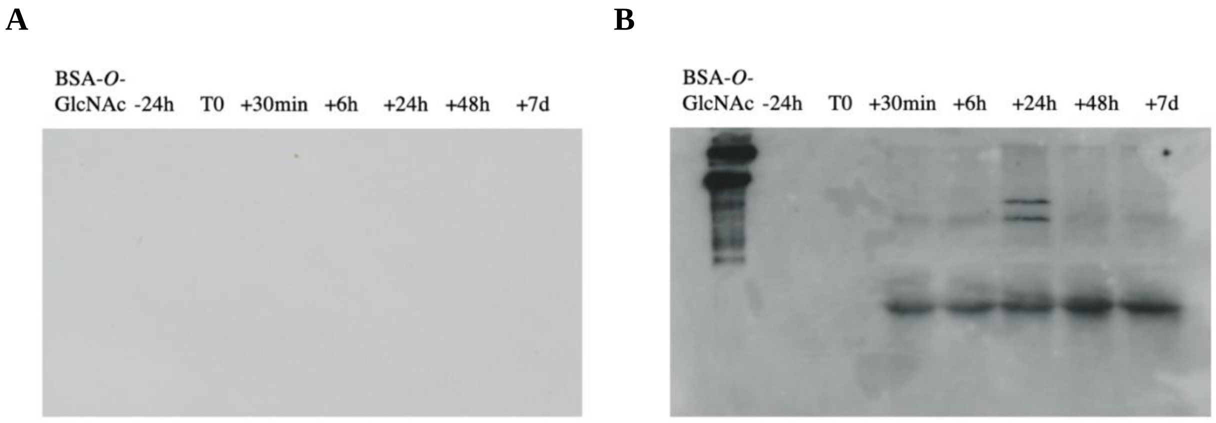

Supplementary Figure 5. Mass spectra of *N*-glycans analysis by MALDI-TOF-TOF. At each sample time, the OS from STIM1 and STIM3 stimulated ewes were pooled.

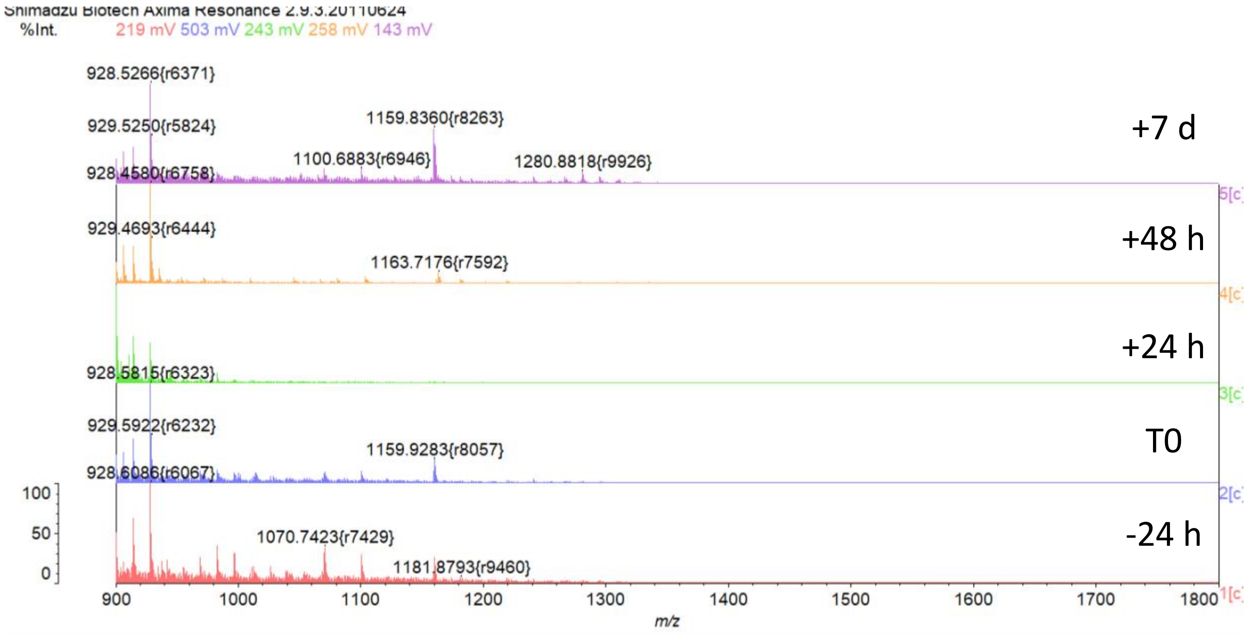

Supplement: Supplementary file 1 [file Data_Sheet_1.PDF]
